# Using a novel genetic algorithm to assess peer influence on willingness to use pre-exposure prophylaxis in networks of Black men who have sex with men

**DOI:** 10.1007/s41109-020-00347-2

**Published:** 2021-03-18

**Authors:** Kara Layne Johnson, Jennifer L. Walsh, Yuri A. Amirkhanian, John J. Borkowski, Nicole Bohme Carnegie

**Affiliations:** 1 Department of Mathematical Sciences, Montana State University, P.O. Box 172400, 59717 Bozeman, MT USA; 2 Center for AIDS Intervention Research, Medical College of Wisconsin, 2071 North Summit Ave., 53202 Milwaukee, WI USA

**Keywords:** Intervention uptake, Opinion diffusion, Parameter estimation, DeGroot model, Genetic algorithm, Pre-exposure prophylaxis (PrEP)

## Abstract

The DeGroot model for opinion diffusion over social networks dates back to the 1970s and models the mechanism by which information or disinformation spreads through a network, changing the opinions of the agents. Extensive research exists about the behavior of the DeGroot model and its variations over theoretical social networks; however, research on how to estimate parameters of this model using data collected from an observed network diffusion process is much more limited. Existing algorithms require large data sets that are often infeasible to obtain in public health or social science applications. In order to expand the use of opinion diffusion models to these and other applications, we developed a novel genetic algorithm capable of recovering the parameters of a DeGroot opinion diffusion process using small data sets, including those with missing data and more model parameters than observed time steps. We demonstrate the efficacy of the algorithm on simulated data and data from a social network intervention leveraging peer influence to increase willingness to take pre-exposure prophylaxis in an effort to decrease transmission of human immunodeficiency virus among Black men who have sex with men.

## Background

The use of interventions for epidemic control requires both an effective intervention and sufficient uptake by the population. In the case of human immunodeficiency virus (HIV), Black men who have sex with men (BMSM) are disproportionately affected by $\text {HIV}$ infection throughout the United States [1]. Pre-exposure prophylaxis (PrEP) has been shown to reduce lifetime infection risk and increase mean life expectancy; however, $\text {PrEP}$ uptake is much lower for $\text {BMSM}$. Negative $\text {PrEP}$-related stereotypes are prevalent and awareness of $\text {PrEP}$ is low among $\text {BMSM}$, particularly those outside of large cities and among men who have sex with men who are not gay-identified nor easily reached through $\text {PrEP}$ campaigns directed toward the gay community [1–3]. An ongoing study seeks to assess the feasibility of increasing $\text {PrEP}$ uptake for $\text {BMSM}$ through the use of a social network intervention: training network leaders to communicate the benefits of $\text {PrEP}$ within their social networks [4]. In this paper, we analyze data from the pilot study of the intervention and present a methodological innovation needed to facilitate evaluation of the possible population-level impact of the intervention on HIV incidence.

Most $\text {BMSM}$ are connected with other men who have sex with men (MSM) of color in their personal social and sexual networks. For that reason, it is possible to reach men through their network connections. In addition to serving as a vehicle for reaching high-risk $\text {BMSM}$ in the community, networks are social environments that can be harnessed for interventions to increase $\text {PrEP}$ awareness, correct $\text {PrEP}$ misconceptions, and strengthen norms, attitudes, benefit perceptions, and skills for $\text {PrEP}$ use. In the $\text {HIV}$ epidemiology literature, the networks of $\text {BMSM}$ have too often been studied only as drivers of disease transmission [5]. However, from a strengths-based perspective, social networks also carry positive, adaptive, and protective functions. $\text {BMSM}$ confront stigma and exclusion due to homophobia in the Black community and racism in predominantly white gay communities, leading some to develop social institutions such as constructed families and house ball communities for support [6–11].

$\text {HIV}$ prevention advice from personally-known and trusted sources is likely to have greater impact than messages from impersonal sources. For that reason, recommendations that come from influential members of one’s close personal social network are especially powerful. Well-liked peers influence the actions and beliefs of their friends. Peers within the close personal networks of $\text {BMSM}$ provide acceptance, trusted information, and guidance on courses of action, including in matters related to $\text {HIV}$ prevention [9]. Messages that provide information but also target the recipient’s $\text {PrEP}$-related perceived norms, attitudes, intentions, and self-efficacy are likely to have the greatest impact because these theory-based domains influence the adoption of protective actions [12, 13].

The intervention is also grounded in principles of innovation diffusion theory [14]. After recruiting networks of $\text {BMSM}$ in the community, the intervention involved selection of a cadre of members within each network who were most socially interconnected with others, most trusted for advice, and most open to $\text {PrEP}$. These network leaders together attended sessions in which they learned about $\text {PrEP}$ and its benefits and were systematically engaged to talk with friends about these topics, correct misconceptions and counter negative stereotypes about $\text {PrEP}$, instill interest in $\text {PrEP}$, and guide interested friends in accessing $\text {PrEP}$ providers. Thus, the intervention engaged trusted and socially-interconnected network leaders to function as agents who diffuse messages to others.

A preliminary analysis on the pilot study data demonstrates more favorable opinions of $\text {PrEP}$ after the network leader intervention, across all subjects and for only those subjects who did not attend leadership training [4]. This analysis supports the continuing use of this intervention from an individual risk perspective. The question remains, however, how impactful the intervention might be if implemented at scale, and how the benefits to HIV prevention compare to similarly intensive interventions. To make this assessment, we plan to use an agent-based epidemic model. In order to evaluate the intervention, it is necessary to translate the results from the pilot and ongoing study to network and intervention parameters in the epidemic modeling framework. For this, we need to understand the structure of influence observed in the local networks. Hence, we wish to estimate the parameters of the opinion diffusion process. Since existing methods for fitting appropriate opinion diffusion models vastly exceed the data available in both the pilot and full study, we developed the parameter estimation method presented here.

We first detail classes of models for opinion diffusion, assessing the appropriateness of each method for modeling the diffusion of opinions about $\text {PrEP}$ through the observed networks and describing our chosen model. We then detail the genetic algorithm we developed to estimate the parameters of the selected opinion diffusion model. Finally, we assess the performance of the algorithm on simulated data and demonstrate its practical application using the pilot study data, discussing behavior of the algorithm and interesting features of the estimated opinion diffusion process.

## Opinion diffusion models

A variety of models exist for *opinion diffusion*: the process through which opinions change and spread through a network. They vary in complexity, underlying assumptions, and the precision or structure of opinions generated. In this section, we outline the main classes of models and justify our choice of the DeGroot model [15] for our intended application.

### Modeling considerations

Our primary considerations for selecting a model are the limited number of time steps available, small observed networks, expected features of $\text {BMSM}$ networks, structure of data collected, and focus on agent-level assessments. The pilot study consisted of observations collected at two time steps (initial opinions and one measurement of opinions in follow-up assessment after the intervention) and the full study will include three time steps (initial opinions and two follow-up assessments). These limited numbers of observations mean an appropriate model will not rely on the system reaching equilibrium. They also limit our ability to estimate a large number of parameters or assess the appropriateness of our selected model using data, informing our preference for simple models that have already been validated on the small networks ($N=4$ to $N=12$) present in the pilot study. Since the networks in the study are themselves clusters from larger networks and contain disinformation, an appropriate model will allow for disinformation under that structure. Because the data consist of Likert-scale measures, the chosen model must make use of the precision available in the data, especially in the absence of more observations. Finally, given our interest in the influence of particular agents within the network, an appropriate model will involve agent-level parameters as opposed to network-level parameters.

### Statistical physics

Statistical physics traditionally focuses on modeling the movement of particles but has increasingly been applied to other fields including opinion diffusion, where agents take the place of particles [16]. Since these models typically involve taking the thermodynamic limit which–in the case of network models–means assuming a network with infinitely many agents, statistical physics models are applied to large networks where each agent interacts with a negligible number of agents relative to the size of the network [17, 18]. Even for networks with hundreds or thousands of agents, this assumption is problematic as behaviors of the diffusion process due to finite size effects are absent from the models using the thermodynamic limit [18]. Given the very small networks included in our data set, models that assume an infinite network are not appropriate. These models also focus on explaining the overall behavior of diffusion process through the actions of individual agents [16, 17] while our goal is to explain the behavior of individual agents through their interactions. Finally, while other candidate models have been validated using data, model validation is largely absent from the statistical physics literature [17].

### $\text {SIR}$ model

Banerjee, Chandrasekhar, Duflo, and Jackson successfully modeled the diffusion of information about microfinance loans between households using a modification of the Susceptible-Infected-Recovered (SIR) epidemic model in which information about microfinance loans takes on the role of the disease [19]. The model was fit to population-level uptake data and does not incorporate agent-level parameters. Though this method would be appropriate for modeling the impact of the intervention on uptake of $\text {PrEP}$ over the limited number of time steps collected, it does not allow for an assessment of how opinions about $\text {PrEP}$ change within the network and would not make full use of the more precise opinion data collected.

### Bayesian and naive learning

Unlike $\text {SIR}$ models, Bayesian learning directly models opinions, rather than uptake; however, these models require distributional assumptions about each agent’s prior belief about the state of the world and the *signals*—or information—received from other agents, both marginally and conditional on the state of the world. Bayesian learning models also assume agents are able to calculate the likelihood of the signals they receive under their current worldview according to Bayes’ rule [20]. These assumptions are problematic both from a modeling perspective and because they imply an unrealistic level of sophistication in the learning mechanisms of each agent. Additionally, this sophistication makes modeling of disinformation difficult [20, 21].

Two different experiments conducted on networks of seven agents using binary signals compared Bayesian learning to non-Bayesian *naive learning*^1^ where agent adopt the majority belief expressed by themselves and their contacts. These experiments demonstrate that naive learning predicts the behaviors of individual agents better than Bayesian learning, especially in highly clustered or insular networks; however, they also indicate that agents behave with more sophistication than is implied by the naive model [21, 22]. Specifically, agents account for dependencies between signals received from agents who are connected to each other, though not to the extent a Bayesian learner would. A slight modification of the naive learning model where agents can place varying importance on the signals of other agents allows for more sophistication in the learning behavior of agents and can even approximate the behavior of a Bayesian learner, especially when the importance can vary with time [22]. This modification brings us to the DeGroot model for opinion diffusion.

### DeGroot model

The DeGroot model is the foundational opinion diffusion model and most influential non-Bayesian model, with the majority of non-Bayesian models being modifications of the DeGroot model [15, 20–22]. Under the DeGroot opinion diffusion model, agents update their opinions at each time step to be a weighted average of their own current opinion and the opinions of everyone with whom they interact. This process is described on a network of *N* agents by

$$\begin{aligned} X (t+1)=WX (t) \end{aligned}$$

where *X* (*t*) is a vector of length *N* with $x_i (t)\in [0,1]$ representing the opinion of agent *i* at time *t* and *W* is an $N\times N$ matrix of weights with $w_{ij}$ representing the weight that agent *i* places on the opinion of agent *j*. The elements in the weight matrix *W* are restricted so that $0\le w_{ij}\le 1$ and $\sum _{j=1}^Nw_{ij}=1$.

*W* is further restricted based on the social network as represented by the adjacency matrix *A*, in which $a_{ij}=a_{ji}=1$ if agents *i* and *j* are connected in the network and $a_{ij}=a_{ji}=0$ otherwise. Since agents can only be directly influenced by the opinions of agents with whom they interact, $w_{ij}=0$ if agents are not connected in the network ($a_{ij}=0$). We set $a_{ii}=1$ to allow agents to update their opinions based on their own current opinions [15]. While we considered extension of the DeGroot model that include bounded confidence or decaying weight placed on the opinions of others, we lack the time steps to assess whether either extension is appropriate or to estimate the relevant parameters. Based on our intended application, the DeGroot model is the clear choice due to its simplicity, ability to model disinformation, capacity for using precise opinion data, and validation on small networks [21, 22].

## Methods

Available methods to estimate parameters of a DeGroot model are quite limited. Castro and Shaikh were able to estimate the parameters of a *Stochastic Opinion Dynamics Model* (SODM)—a variant of the DeGroot model which includes information from external sources and normal measurement error on observed opinions—using data collected from an observed opinion diffusion process over an online social network. While they developed both maximum-likelihood-based and particle-learning-based algorithms for estimating the parameters of the $\text {SODM}$, these algorithms require more time steps than agents and at least 100 time steps, respectively [23, 24]. Since the requirements for the existing algorithms vastly exceed the available data for the $\text {PrEP}$ studies and similar opinion diffusion applications, we developed a novel genetic algorithm capable of recovering the parameters of a DeGroot opinion diffusion process—the elements in the weight matrix *W*—using small data sets, including those with missing data and more model parameters than observed time steps.

### Objective function

We propose a squared deviation function summed across all *N* agents and *T* time steps^2^ which measures how closely the predicted opinions match the observed opinions:

$$\begin{aligned} f_C ({\hat{X}},X)=\sum _{i=1}^N\sum _{t=0}^{T-1}\big ({\hat{x}}_i (t)-x_i (t)\big )^2. \end{aligned}$$

Since lower values indicate a better fit, the optimal solution is one that minimizes the value of the objective function. To assess fit at the agent-level, we simply exclude the sum over all agents and instead compute a separate value of the objective function for each agent using

$$\begin{aligned} f_C ({\hat{x}}_i,x_i)=\sum _{t=0}^{T-1}\big ({\hat{x}}_i (t)-x_i (t)\big )^2. \end{aligned}$$

### Genetic algorithm

Genetic algorithms, which mimic the natural processes through which the fittest genes survive to subsequent generations, are an ideal choice for fitting a DeGroot model; they have fewer assumptions and a lower chance of becoming stuck in local optima compared to other optimization algorithms [25]. Genetic algorithms consist of a population of *chromosomes*, or complete solutions to the optimization problem, which are each composed of *genes*, subsets of the solution consisting of either individual values or collections of values. In each iteration of the algorithm, these parent chromosomes undergo a variety of operators which modify the genes, producing a population of offspring chromosomes that differ from the population of parent chromosomes. This process repeats with the offspring chromosomes becoming the parent chromosomes of the subsequent iteration until some stopping criterion is met [25]. In the case of the simple DeGroot model, a chromosome is defined as the weight matrix *W* and a gene as a single row of the weight matrix, denoted $W_i$. This means each gene corresponds to an individual agent and represents the weights that agent places on the opinions of other agents.

We adapt a genetic algorithm developed for generating *D*-optimal designs for constrained mixture experiments by Limmun, Borkowski, and Boonorm: a useful starting point since both the design matrix for mixture experiments and the weight matrix for a DeGroot model are row-stochastic with the elements in each gene summing to 1 [25]. Though this common constraint on the matrices means the operators developed for mixture experiments are well suited for estimating the parameters of the DeGroot model, there is an important difference in the way the objective functions are used to assess the fitness of chromosomes and the genes within them. Under *D*-optimality, the fitness of a gene within the design matrix is dependent on the other genes and cannot be assessed separately from the fitness of the entire chromosome. In contrast, since each gene corresponds to an agent, the objective function for a DeGroot opinion diffusion process can be assessed at the gene-level by assessing how well the model predicts the opinions of a particular agent, as demonstrated in the above objective function. We leverage this ability to assess fit on the gene-level by incorporating a gene-swapping process into the algorithm along with the selection, blending, crossover, mutation, and survival operators described below.

#### Gene-swapping

While the objective function can be assessed at the agent or gene-level, since agents update their opinions based on the opinions of others, the fitness of a gene is dependent on the predicted opinions of the other agents which are, in turn, dependent on the other genes within the chromosome. Consider a case where agent 1 has an unfavorable view of $\text {PrEP}$ which changes to a favorable view after speaking with their contacts, including agent 2. A gene corresponding to agent 1 which places high weight on agent 2 will fit well if the model predicts a favorable view for agent 2 but fit poorly if the model predicts an unfavorable view. In essence, when presented with a pair of genes corresponding to the same agent between two chromosomes, it is possible to assess which gene better predicts the opinions of the agent within its current chromosome but the fitter gene is not guaranteed to continue to produce better predicted opinions when swapped with a less fit gene in an otherwise fitter chromosome.

For example, consider the following population of chromosomes (*B*, *C*, and *D*) for a network of $N=3$ agents whose opinions are recorded across $T=6$ time steps. The value of the objective function for each chromosome, broken down by gene, is given to the right of the chromosome. Genes of interest, along with their contributions to the value of the objective function, are bolded.

$$\begin{aligned} B= & {} \begin{bmatrix} 0.336 &{} 0.664 &{} 0.000\\ \mathbf{0.261 } &{} \mathbf{0.364 } &{} \mathbf{0.375 }\\ 0.349 &{} 0.306 &{} 0.345 \end{bmatrix}, \quad f ({\hat{X}}_B,X)=0.125+ \mathbf{0.017 }+0.006=0.148 \\ C= & {} \begin{bmatrix} 0.378 &{} 0.622 &{} 0.000\\ 0.509 &{} 0.324 &{} 0.167\\ \mathbf{0.441 } &{} \mathbf{0.381 } &{} \mathbf{0.178 } \end{bmatrix}, \quad f ({\hat{X}}_C,X)=0.093+0.036+ \mathbf{0.001 }=0.130 \\ D= & {} \begin{bmatrix} 0.845 &{} 0.155 &{} 0.000\\ \mathbf{0.235 } &{} \mathbf{0.427 } &{} \mathbf{0.338 }\\ \mathbf{0.349 } &{} \mathbf{0.306 } &{} \mathbf{0.345 } \end{bmatrix}, \quad f ({\hat{X}}_D,X)=0.007+ \mathbf{0.044 }+ \mathbf{0.005 }=0.056 \end{aligned}$$

Compared to chromosome *D*, the second gene in chromosome *B* and the third gene in chromosome *C* produce predicted opinions closer to the observed opinions (objective function contributions of $0.017<0.044$ and $0.001<0.005$); however, when the fitter genes are swapped with the corresponding genes in chromosome *D*, the value of the objective function for the new chromosome $D^*$ increases, indicating the swapped genes perform worse than the original genes within chromosome *D*. This is due to changes in the predicted opinions over time when modified weights are placed on others’ opinions.

$$\begin{aligned} D= & {} \begin{bmatrix} 0.845 &{} 0.155 &{} 0.000\\ \mathbf{0.235 } &{} \mathbf{0.427 } &{} \mathbf{0.338 }\\ \mathbf{0.349 } &{} \mathbf{0.306 } &{} \mathbf{0.345 } \end{bmatrix}, \quad f ({\hat{X}}_D,X)=0.056 \\ D^*= & {} \begin{bmatrix} 0.845 &{} 0.155 &{} 0.000\\ \mathbf{0.261 } &{} \mathbf{0.364 } &{} \mathbf{0.375 } \\ \mathbf{0.441 } &{} \mathbf{0.381 } &{} \mathbf{0.178 } \end{bmatrix}, \quad f ({\hat{X}}_D^*,X)=0.071 \end{aligned}$$

Within the algorithm, the gene-swapping process behaves in a manner very similar to the above example. The fittest chromosome, which we again call *D*, is identified and the fitness of each chromosomes is assessed at the gene-level. We then compare the fitness of each gene within *D* to the fitness of the corresponding genes in all other chromosomes. In all cases where a fitter gene is present, the corresponding genes are swapped between *D* and the chromosomes containing fitter genes, resulting in $D^*$ and modified versions of all other chromosomes previously containing fitter genes. After all swaps are completed, we compare the fitness of *D* to the fitness of $D^*$. All swaps are retained if $D^*$ is the fitter chromosome and all swaps are rejected, returning all chromosomes to their previous version, if *D* is the fitter chromosome.

#### Selection

The purpose of the selection operator is to preserve the current optimal solution if the subsequent iteration of the algorithm is unable to find a better one. To this end, we implement *selection with elitism*, where the best chromosome is identified prior to each iteration of the algorithm. This elite chromosome undergoes gene swapping with non-elite chromosomes and then remains unchanged for the remainder of the current iteration of the algorithm, while the remaining chromosomes undergo the subsequent operators.

#### Blending

For blending, all non-elite chromosomes are randomly paired. Blending occurs for each pair of genes between paired parent chromosomes independently with probability $p_b$ and produces new genes which are weighted averages of the chosen gene pairs. Suppose chromosomes *B* and *C* are paired and that $B_i$ and $C_i$ are the *i*th genes in chromosomes *B* and *C*, respectively. Assuming blending occurs for row *i*, the offspring genes $B^*_i$ and $C^*_i$ are defined as

$$\begin{aligned} B^*_i=\beta B_i+ (1-\beta )C_i\quad \text {and}\quad C^*_i= (1-\beta )B_i+\beta C_i \end{aligned}$$

where $\beta \sim Unif (0,1)$ is a randomly selected blending factor.

#### Crossover

We use a modified version of the within-parent crossover operator developed by Limmun, Borkowski, and Boonorm [25]. Modifications are required to adequately explore the relatively large parameter space of the DeGroot model, in contrast to the smaller design space in constrained mixture experiments. Within-chromosome crossover occurs with probability $p_c$ independently for all genes in the non-elite chromosomes. For genes selected for crossover, all non-fixed weights are randomly permuted within the gene.

#### Mutation

Mutation of a gene occurs with probability $p_m$ independent of all other genes in the non-elite chromosomes. For mutation, a weight *w* is selected randomly from all non-fixed weights within the gene and a random perturbation $\varepsilon \sim N (0,\sigma ^2)$ is added to the selected weight to produce $w^*=w+\varepsilon$. All other non-fixed weights within the row are then scaled by $\frac{1}{1-w_{fixed}-w^*}$, where $w_{fixed}$ is the sum of all fixed weights within the row, so that the row sums to 1. We provide more details on fixed weights below in the *Other Features* section. To avoid division by zero and maintain weights in the interval [0,1], we implement the following special cases:

- If $w^*<0$, $w^*$ is set to 0
- If $w^*>1-w_{fixed}$, $w^*$ is set to $1-w_{fixed}$. All other non-zero weights in the row are then set to 0.
- If the selected weight $w=1-w_{fixed}$, the excess weight of $1-w_{fixed}-w^*$ is evenly distributed between all other non-fixed weights within the row.

#### Survival

The survival operator is a means to retain only offspring chromosomes that are an improvement over the corresponding parent chromosomes. The survival operator occurs after mutation, blending, and crossover and compares each pair of parent and offspring chromosomes and retains the fitter of each pair. The retained chromosomes are then subject to gene swapping with their corresponding rejected chromosomes. The final retained chromosomes become the parent chromosomes for the subsequent iteration of the algorithm.

#### Other features

The fixed values mentioned in the mutation operator allow for specification of elements in the weight matrix *W* whose values are either known or assumed. Most often, this will be because weights are fixed at 0 because agents are not linked in the network ($w_{ij}$ where $a_{ij}=0$).

For early iterations of the algorithm, the purpose of the crossover and mutation operators is to explore the parameter space of the DeGroot model. To this end, we use relatively high values of the probabilities ($p_c,p_m$) and of $\sigma$. In later iterations, the purpose shifts to refining an existing solution. Since the blending operator is better suited to this purpose, we progressively reduce the probabilities of crossover and mutation and the mutation variance $\sigma ^2$ while increasing the probability of blending ($p_b$). These changes are implemented using a multiplicative adjustment each time a specified number of iterations with no improvement is reached (until the probabilities and $\sigma$ attain specified minimum or maximum values). The hyperparameters of the algorithm used in both the simulation study and data analysis are given in Table 1. The ranges of operator probabilities ($p_b,p_c,p_m$) and $\sigma$ are informed by the work of Limmun, Borkowski, and Boonorm [25] with adjustments to accommodate searching a larger space. The remainder of the parameters were selected based on our experience using the algorithm to heavily favor convergence over computational efficiency with the intention of the values having minimal impact on results.

**Table 1:** Simulation study hyperparameters

Hyperparameter	Value	Description
chromosomes	21	Number of chromosomes
probb	0.01	Initial probability of blending ($p_b$)
factorb	2	Multiplicative factor for modifying $p_b$
maxb	0.2	Maximum value of $p_b$
iterb	1000	Number of iterations with no improvement before modifying $p_b$
probc	0.2	Initial probability of crossover ($p_c$)
factorc	0.5	Multiplicative factor for modifying $p_c$
minc	0	Minimum value of $p_c$
iterc	1000	Number of iterations with no improvement before modifying $p_c$
probm	0.2	Initial probability of blending ($p_m$)
factorm	0.5	Multiplicative factor for modifying $p_m$
minm	0.01	Minimum value of $p_m$
iterm	1000	Number of iterations with no improvement before modifying $p_m$
sigma	1	Initial value of standard deviation $\sigma$ of error for mutation operator
factors	0.5	Multiplicative factor for modifying $\sigma$
mins	0.001	Minimum value of $\sigma$
iters	2000	Number of iterations with no improvement before modifying $\sigma$
max_iter	1E6	Maximum number of iterations to run algorithm
min_improve	0	Minimum decrease in value of objective function considered an improvement
min_dev	0	Acceptable value of objective function for stopping algorithm
reintroduce	“elite”	type of chromosome to be reintroduced
iterr	2500	Number of iterations with no improvement before reintroducing chromosome

The table gives the name of each hyperparameter in the software developed to implement the new genetic algorithm, the value used for the simulation study and data analysis in the paper, and a description of the hyperparameter

We also implement a chromosome reintroduction process that serves either to support a prior belief that agents will place high weight on their own opinions or to aid the refinement process. In either case, after a specified number of iterations without improvement, the worst chromosome—as identified by the highest value of the objective function—is removed from the population and replaced with a reintroduced chromosome. To support a prior belief about high self-weight, the reintroduced chromosome has $1-w_{fixed}$ along the diagonal and zero for all other non-fixed weights. To aid the refinement process, the reintroduced chromosome is a clone of the elite chromosome which would otherwise be exempted from the the remaining operators in the selection operator. We reintroduce the elite chromosome in the simulation study and data analysis for this paper. A chromosome with $1-w_{fixed}$ on the diagonal is always included in the population of initial chromosomes.

## Simulation study

We first demonstrate the performance of the algorithm implemented in Julia [26] on simulated data varying the factors described in Table 2. The values chosen are informed by the intended application of the algorithm to the pilot and full $\text {PrEP}$ studies as well as possible features of other network studies ill-suited to existing methods: those with few observations per agent on smaller networks. Since the smallest network in the pilot study had four agents, we use $N=4$ as the smallest network in the simulation study. Though data collection methods that can practically be applied to networks of 50 agents should allow for the collection of a sufficient number of time steps to use alternate algorithms, we include a network of size $N=50$ to ensure the range of network sizes considered includes all networks where this algorithm is the most practical option.

The use of number of time steps of $T=2$ and $T=3$ are based on the number of time steps in the pilot and full study, respectively. While the motivation for the development of this method is the ability to fit opinion diffusion models using small data sets, the number of time steps available in the $\text {PrEP}$ studies are extremely small. We include time steps of $T=6$ and $T=11$ (initial opinions plus five and ten follow-up assessments, respectively) in order to assess the impact of these extremely small samples on algorithm performance compared to other small samples. Though at most $T=11$ time steps are ever provided to the algorithm, we simulate data over 21 time steps so that the time steps provided to the algorithm and the time steps withheld would serve as pseudo testing and training sets, allowing us to assess the ability of the algorithm to project opinions past the time steps on which data were collected. We use 21 time steps so that the number of time steps over which we extrapolate is the same as the number of time steps past initial provided to the algorithm for the largest number of time steps used ($T=11$).

**Table 2:** Simulation study inputs

Input	Values	Notes
Network size	$N=4,10,20,50$	Reachability enforced
Degree	$d=2,5,9$	Minimum degree $d=1$ for all nodes
Self-weight	$w_{ii}=0.1,0.5,0.8$	Beta distribution with $\kappa =\alpha +\beta =10$
Time steps	$T=2,3,6,11$	Performance assessed on $t=1,\ldots ,20$
Missingness	$0\%,10\%,25\%,50\%$	Minimum 2 observations per agent

Values for factors used in the simulation study

In each simulation, we generate an Erdős–Rényi random network of size *N* with with connection probability $p=\frac{d}{N-1}$ based on a target degree of *d*, excluding mathematically impossible combinations. While the overall structure of the social networks being leveraged is unlikely to be a random network, the use of relatively dense random networks reflects the attempt to sample clusters in the $\text {BMSM}$ social network for intervention. We enforce reachability between all pairs of nodes by rejecting any generated networks that are not connected. This rejection does inflate the average degree beyond the target with the inflation being worse for smaller degrees as seen in Table 3. We address this in our analysis of the simulation study results by using the observed degree in place of the categorical target degree where reasonable. We use the same approach with self-weight, though the mean self-weight within each target category is equal to the target when rounded to two decimal places.

**Table 3:** Target and mean degrees

Target	Mean
$d=2$	${\bar{d}}=2.47$
$d=5$	${\bar{d}}=5.03$
$d=9$	${\bar{d}}=9.01$

Target degree and observed mean degree from simulation study

We then randomly generate a weight matrix subject to a target self-weight. Each $w_{ii}$ is drawn from a beta distribution with concentration $\kappa =\alpha +\beta =10$ with $\alpha$ and $\beta$ derived from the concentration and the target mean. Weights are fixed as indicated by the adjacency matrix of the social network so that $w_{ij}=0$ if $a_{ij}=0$. The remaining $1-w_{ii}$ in each row is randomly distributed between all weights not fixed at 0. This weight matrix is then used to simulate the opinion diffusion process according to the DeGroot model over twenty time steps past the initial (total of 21). Initial opinions are drawn from a *Unif* (0, 1) distribution. Finally, we create the “observed” data set by restricting to *T* time steps and randomly removing a specified percentage of observations, ensuring that no initial observations were missing and at least one other observation is non-missing for each agent. Again, combinations of network size and missing data which are incompatible with the above requirements are excluded. We then use the algorithm to fit the DeGroot model to the simulated data set, repeating the process ten times for each generated data set.

Performance is assessed for correct prediction of opinions across the time steps used to fit the weight matrix (*T*), correct prediction of opinions across the time steps extrapolated past those used to fit the weight matrix ($21-T$), and recovery of weights. We used the root-mean-square error (RMSE) for all assessments:

$$\begin{aligned} {RMSE}_{fit}= & {} \sqrt{\frac{\sum _{t=0}^{T-1}\sum _{i=1}^N \big ({\hat{x}}_i (t)-x_i (t)\big )^2}{N (T-1)}}, \\ {RMSE}_{ext}= & {} \sqrt{\frac{\sum _{t=T}^{21}\sum _{i=1}^N \big ({\hat{x}}_i (t)-x_i (t)\big )^2}{N (21-T)}}, \end{aligned}$$

and

$$\begin{aligned} {RMSE}_{rec}=\sqrt{\frac{\sum _{i=1}^N\sum _{j=1}^N (w_{ij}-{\hat{w}}_{ij})^2}{\sum _{i=1}^N\sum _{j=1}^Na_{ij}}}=\sqrt{\frac{\sum _{i=1}^P (w_p-{\hat{w}}_p)^2}{P}}, \end{aligned}$$

where *P* is the number of elements not fixed at zero in the weight matrix (the number of parameters to be estimated) and $w_p$ is the *p*th non-zero element with $w_p$ and ${\hat{w}}_p$ representing the true and estimated weights, respectively.

### Results

Since possible proportions of missing data depend on the number of time steps and possible degrees depend on the network size, we assess the effect of each pair of variables together. We also make use of the mean number of observations per agent, which incorporates information about both missingness and time steps. For each variable or combination of variables, we assess the ability of the algorithm to recover the model parameters and investigate whether high recovery RMSE is the result of the algorithm identifying a solution that fits the data poorly or identifying a solution that fits the data well without recovering the model parameters. We also explore whether fit on the time steps used for estimation is indicative of fit on time steps extrapolated past those used to estimate the weight matrix. Because RMSE is bounded below and many of the plots used show right-skew, we present all summary statistics for RMSE using median and IQR.

#### Network size and degree

**Fig. 1:**
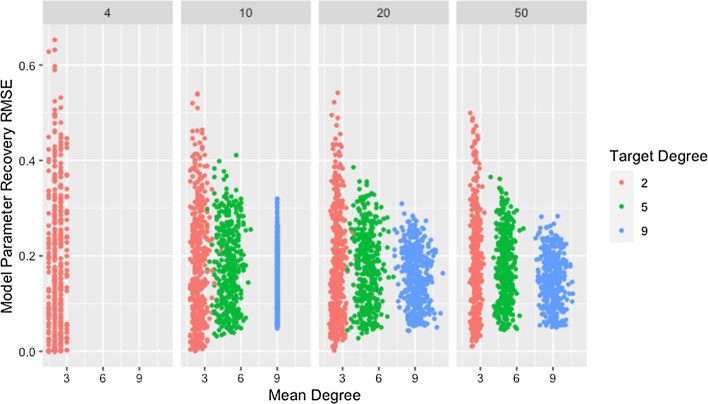
RMSE for model parameter recovery by degree and size. RMSE for known and predicted weights by mean degree, target degree, and network size across panels

Figure 1 and Table 4 show a clear decrease in variability of the RMSE for weight recovery, with increasing degree and network size. They also indicate that recovery tends to improve with increasing degree and size, though the differences are small and this relationship is inconsistent for low degree networks. As noted previously, the method for generating networks inflates the actual degree beyond the target for low-degree networks. The effect is worse for larger networks as indicated in Fig. 1 and confirmed by Table 5. The high variability for low degree networks combined with the observed degree being inflated beyond the target may explain the trend in observed medians for networks with a target degree of $d=2$.

**Table 4:** Median recovery RMSE by degree and sizee

Size	Degree		
	2	5	9
4	0.15 (0.23)	NA	NA
10	0.17 (0.18)	0.17 (0.14)	0.15 (0.10)
20	0.17 (0.17)	0.18 (0.12)	0.15 (0.10)
50	0.17 (0.15)	0.17 (0.12)	0.15 (0.08)

Median and IQR of RMSE for model parameter recovery by degree and network size

**Table 5:** Mean degree by size

Size	Mean
4	${\bar{d}}=2.15$
10	${\bar{d}}=2.48$
20	${\bar{d}}=2.58$
50	${\bar{d}}=2.62$

Observed mean degree from simulation study for networks with target degree of $d=2$ by network size

Initially, both of these relationships seem counter-intuitive since increasing either network size or degree increases the number of model parameters to be estimated. We propose that the observed relationship is due to the dependencies between weights within a row, resulting from the restriction that the weights must sum to one. For a network where agents have a degree of two, there are at most three non-zero weights within each row. If one of these weights differs from the true weight, either one or both of the others must also differ from the true weight, inflating the recovery RMSE. As the degree increases, if one weight differs, it becomes possible for other weights in the row to closely match the truth since the excess weight can be distributed between or taken from the many other weights within the row. For networks with low degree, once a single weight within a row differs, this effect cascades through the rest of the row and produces high recovery RMSE, explaining both the higher median and variability for networks with low degree.

**Fig. 2:**
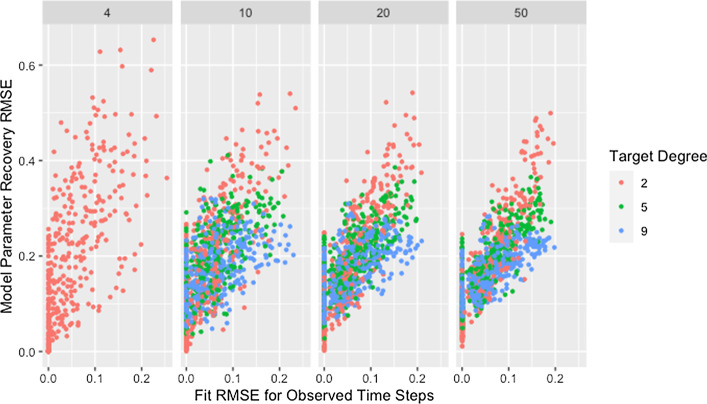
RMSE for model parameter recovery by fit RMSE, degree, and size. RMSE for known and predicted weights by RMSE for observed and predicted opinions on observed time steps, target degree, and network size across panels

This explanation does not address whether the algorithm struggles to identify good solutions for networks with low degree since the cascading effect occurs whether or not these weights produce predicted opinions close to those observed. In fact, we would expect model parameter recovery to be easiest for low-degree networks since there are fewer model parameters to recover. This is supported by the presence of near perfect model parameter recovery for small networks with low degree as demonstrated in Fig. 1 and Table 6. Figure 2 confirms that these low-degree networks with high recovery RMSE are not the result of the algorithm failing to identify an adequate solution but are instead the result of solutions that fail to recover the model parameters while still predicting opinions with reasonable accuracy. This is especially true for small networks (which we consider in more detail below).

**Table 6:** Minimum recovery RMSE by degree and size

Size	Degree		
	2	5	9
4	$4.92\times 10^{-8}$	NA	NA
10	$3.89\times 10^{-4}$	$1.68\times 10^{-3}$	$1.05\times 10^{-2}$
20	$3.17\times 10^{-2}$	$2.75\times 10^{-2}$	$4.48\times 10^{-2}$
50	$4.77\times 10^{-2}$	$4.29\times 10^{-2}$	$5.00\times 10^{-2}$

Minimum RMSE for model parameter recovery by target degree and network size

**Fig. 3:**
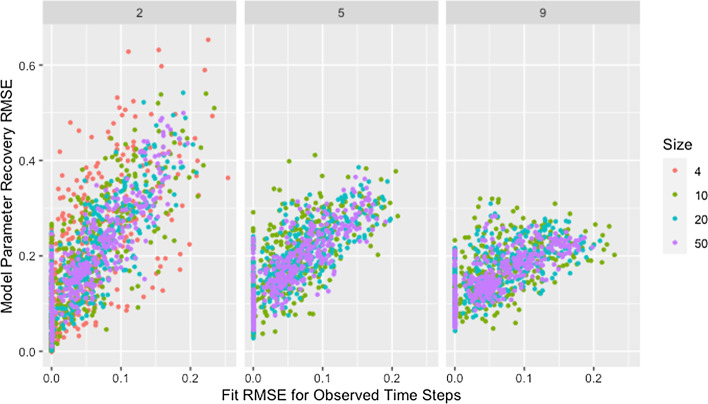
RMSE for model parameter recovery by fit RMSE, size, and degree. RMSE for known and predicted weights by RMSE for observed and predicted opinions on observed time steps, network size, and target degree across panels

Though the effect of network size is slightly complicated by self-weight—as we will discuss later—we postulate that the decrease in median RMSE and IQR for larger networks is a result of the larger networks mitigating the dependency induced between weights by the degree of each agent. While low degree ensures that a single poorly recovered weight will result in more poorly recovered weights within the row, in larger networks that problematic row has less influence on other rows. This is supported by Fig. 3, which demonstrates that—within a target degree—larger networks result in less variability in model parameter recovery for similar values of fit RMSE. This figure also confirms that the higher median and variability in recovery RMSE for smaller networks is not the result of the algorithm failing to identify a solution that fits the data, since the marginal distribution of fit RMSE is comparable across network sizes.

#### Self-weight

**Fig. 4:**
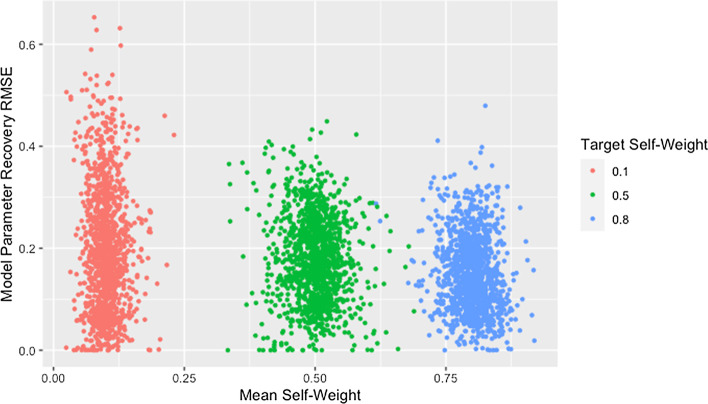
RMSE for model parameter recovery by self-weight. RMSE for known and predicted weights by observed mean self-weight and target self-weight

Figure 4 and Table 7 demonstrate that lower self-weights are more difficult to recover and produce greater variability in the RMSE. We theorize that this discrepancy is partially explained by the fact that the same target self-weight was used for all agents. Agents with high self-weight necessarily place low weight on the opinions of other agents, so their predicted opinions will remain fairly stable over time and as the opinions of other agents change during estimation. As a result, the fit of a row with high self-weight will be robust to incorrect predicted opinions of other agents, implying robustness to incorrect estimated weights for other agents. The opposite is true for agents with low self-weight: the fit of a row is highly dependent on the estimated weights of other agents and the predicted opinions they generate, making estimation more unstable.

**Table 7:** Median recovery RMSE by self-weight

Target self-weight	${\mathbf{RMSE}}_{{{\mathbf{rec}}}}$
0.1	0.18 (0.18)
0.5	0.17 (0.12)
0.8	0.14 (0.10)

Median and IQR of RMSE for model parameter recovery by target self-weight

**Fig. 5:**
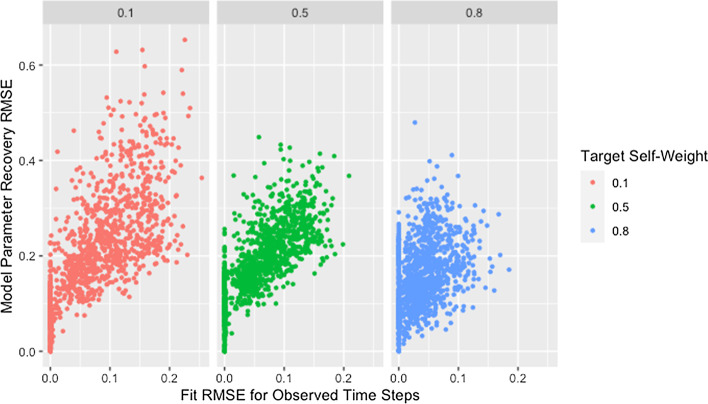
RMSE for model parameter recovery by fit RMSE and self-weight. RMSE for known and predicted weights by RMSE for observed and predicted opinions on observed time steps and target self-weight

**Fig. 6:**
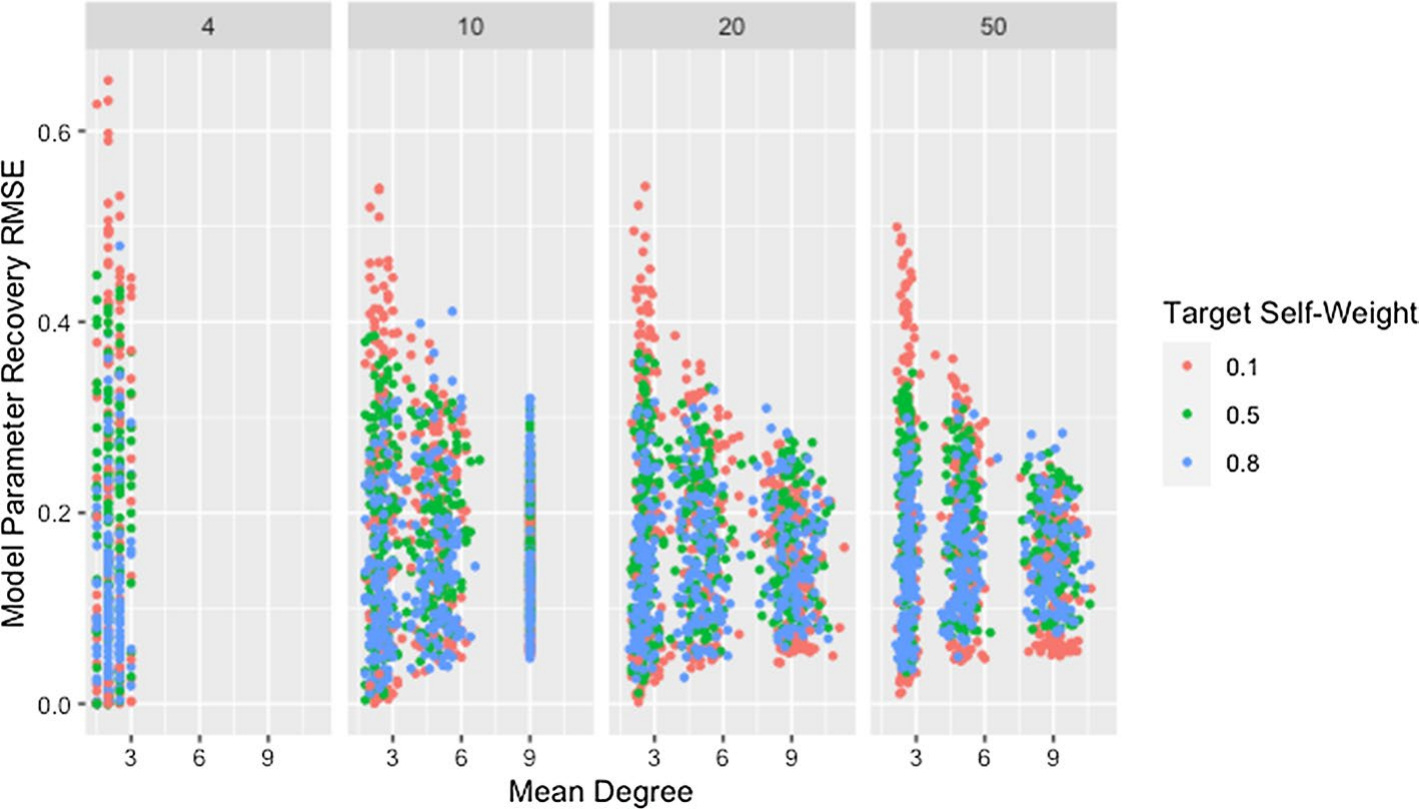
RMSE for model parameter recovery by degree, self-weight, and size. RMSE for known and predicted weights by mean degree, target self-weight, and network size across panels

However, Fig. 5 demonstrates that networks with high recovery RMSE do not have particularly high fit RMSE, implying that the above explanation alone does not explain the patterns observed. We suggest that the high recovery RMSE for networks with low self-weight is caused by a similar phenomenon as was suggested in our discussion of the effect of degree: the strong dependencies between rows within networks with low self-weight result in a single incorrect weight affecting the rows for agents on whom incorrect weight is placed. Including self-weight in our assessment of size and degree supports this idea as demonstrated by Fig. 6, which shows all of the highest recovery RMSE values are from networks with low self-weight. This is consistent with the hypothesized effects of both degree and self-weight. When a single weight within a row of low degree is incorrect, the other weights in the row must also be incorrect. When agents have low self-weight, these incorrect weights in a single row spread to other rows which must also contain multiple incorrect weights because of the low degree. As the geodesic distance between the agent with incorrect weights and other agents increases, the rows corresponding to those other agents become progressively less influenced by the incorrect weights. This allows size to mitigate the effect of low self-weight as we suggested it did for low degree.

#### Missingness and time steps

**Fig. 7:**
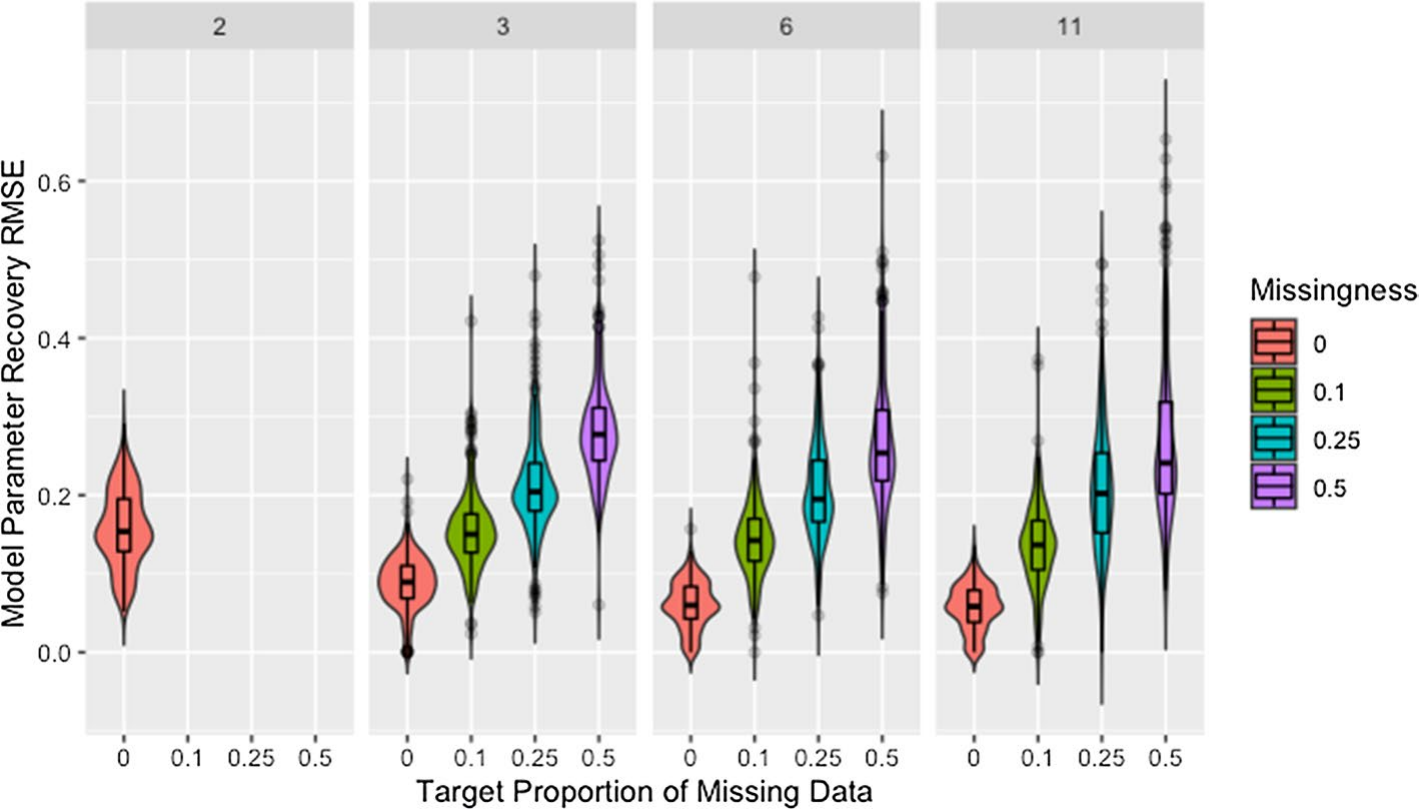
RMSE for model parameter recovery by missingness and time steps. RMSE for known and predicted weights by proportion of missing data past initial and number of time steps across panels

**Fig. 8:**
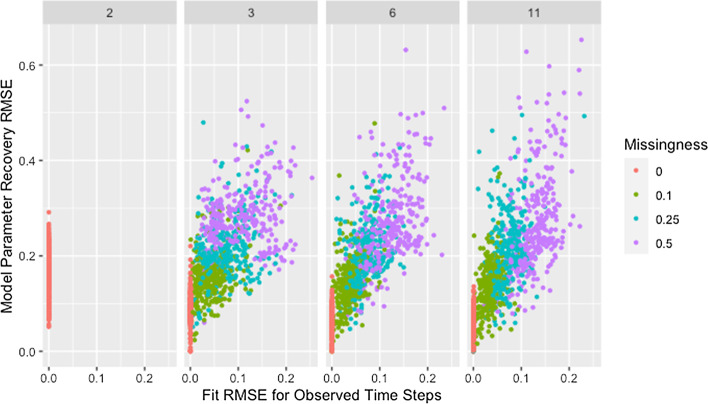
RMSE for model parameter recovery by fit RMSE, missingness, and time steps. RMSE for known and predicted weights by RMSE for observed and predicted opinions on observed time steps, proportion of missing data, and number of time steps across panels

Figure 7 shows model parameter recovery improves with more time steps of data used for estimation and lower proportions of missing data, though both Fig. 7 and Table 8 indicate diminishing improvement as the number of time steps increases. While Table 8 also demonstrates that variability in RMSE tends to increase with more time steps and higher proportions of missing data, it is unclear if this relationship holds for low proportions of missing data. Figure 8 confirms the decreased recovery ability for lower numbers of time steps is not the result of poor fit on the time steps used for estimation, instead the ability of the algorithm to recover parameters is improved when more data are available, as is to be expected.

**Table 8:** Median recovery RMSE by rate of missingness and time steps

Time steps	Missingness			
	0	0.1	0.25	0.5
2	0.15 (0.07)	NA	NA	NA
3	0.09 (0.04)	0.15 (0.05)	0.20 (0.06)	0.28 (0.07)
6	0.06 (0.04)	0.14 (0.05)	0.19 (0.08)	0.25 (0.09)
11	0.06 (0.04)	0.14 (0.06)	0.20 (0.10)	0.24 (0.12)

Median (IQR) of RMSE for model parameter recovery by proportion of missing data and number of time steps used for estimation

**Fig. 9:**
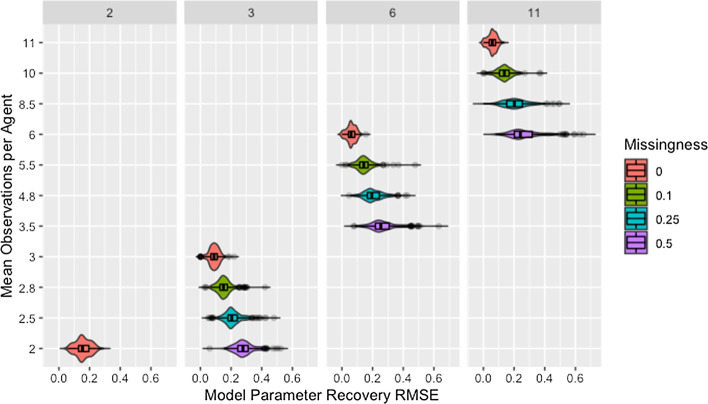
Observations by RMSE for model parameter recovery, missingness, and time steps. Mean number of observations per agent by RMSE for known and predicted weights, proportion of missing data past initial, and number of time steps across panels

Though the relationships between model parameter recovery and both missingness and time steps are intuitive in that fewer observations result in worse recovery of model parameters, whether the presence of missing data is problematic solely because it reduces the amount of information available for fitting the model is unclear from the information in either Fig. 7 or Table 8. To address this, we present Fig. 9, which demonstrates that, for a given mean number of observations per agent, the algorithm is more accurate and precise when provided with more complete data from fewer time steps (see also Table 9). We suggest this is due to the data being missing at random instead of distributed evenly between agents or across time points, making some observations more valuable than others.

**Table 9:** Median recovery RMSE by missingness and observations

Observations	Missingness	
	0	0.5
2	0.15 (0.07)	0.28 (0.07)
6	0.06 (0.04)	0.24 (0.12)

Median and IQR of RMSE for model parameter recovery by proportion of missing data and mean number of observations per agent

Figure 9 confirms that the distribution of missing data between time steps is an issue based on the comparison of networks with two time steps to those with three time steps past initial and 50% missing data. Based on the requirement that all agents have at least one non-missing observation past initial, all agents in networks with three time steps and 50% missing data have two observations but the second observation could occur at either the first or second time step. While both setups result in the same number of observations per agent, recovery RMSE is higher for the networks with missing data, indicating missing data increases recovery RMSE beyond what would be expected from the decreased number of observations per agent.

**Fig. 10:**
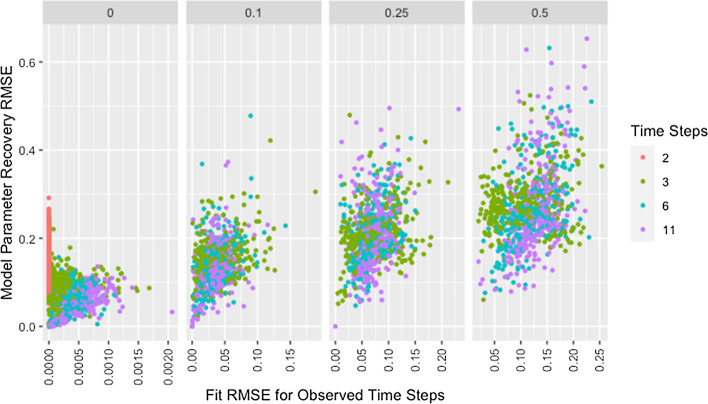
RMSE for model parameter recovery by fit RMSE, time steps, and missingness. RMSE for known and predicted weights by RMSE for observed and predicted opinions on observed time steps, number of time steps, and proportion of missing data across panels with different x-axis scales

**Fig. 11:**
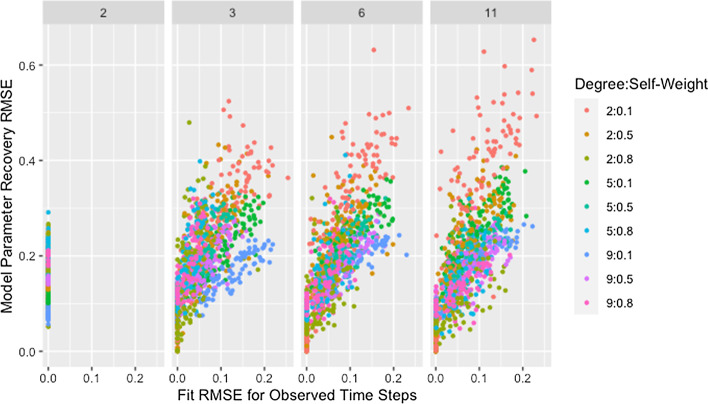
RMSE for model parameter recovery by fit RMSE, degree, self-weight, and time steps. RMSE for known and predicted weights by RMSE for observed and predicted opinions on observed time steps, target degree, target self-weight, and number of time steps across panels

As shown in Fig. 10, with the exception of simulations with only two time steps, recovery RMSE roughly increases with fit RMSE and larger values for both measures tend to be from networks with more missing data. The lack of a relationship for simulations with two time steps indicates fit on the observed time steps is a poor indicator of parameter recovery when only two time steps are available. While Fig. 8 also indicates the presence of unusually high recovery RMSE relative to fit RMSE for some networks with high missingness, high missingness alone does not explain this. In Fig. 11 we can see that all such networks have either low self-weight, low degree, or both.

**Fig. 12:**
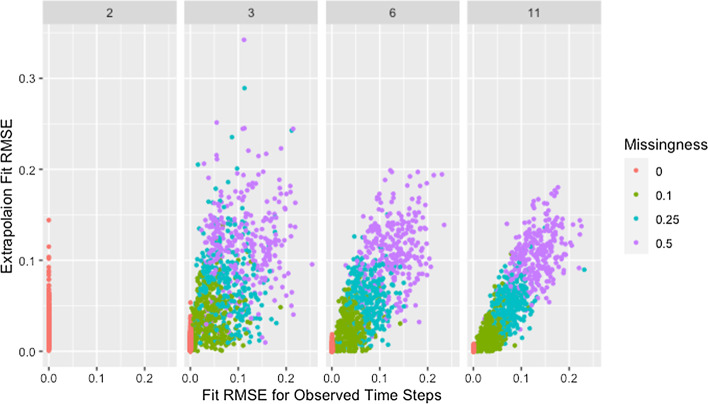
Extrapolation RMSE by fit RMSE, missingness, and time steps. RMSE for observed and predicted opinions on extrapolated time steps by RMSE for observed and predicted opinions on observed time steps, proportion of missing data, and number of time steps across panels

We see a similar pattern with fit on the observed time steps and fit on extrapolated time steps where networks with only two time steps behave differently than all others. Figure 12 shows the relationship between fit on the time steps provided to the algorithm (observed time steps) and fit on the time steps not provided to the algorithm (extrapolated time steps). With the exception of simulations where only two time steps are provided to the algorithm, fit on the observed time steps is indicative of fit on extrapolated time steps with the strength of this relationship increasing with an increasing number of time steps provided to the algorithm. Since RMSE for the time steps provided to the algorithm ignores the presence of missing data, Fig. 12 includes clusters with higher $RMSE_{fit}$ and $RMSE_{ext}$ for higher proportions of missingness.

**Fig. 13:**
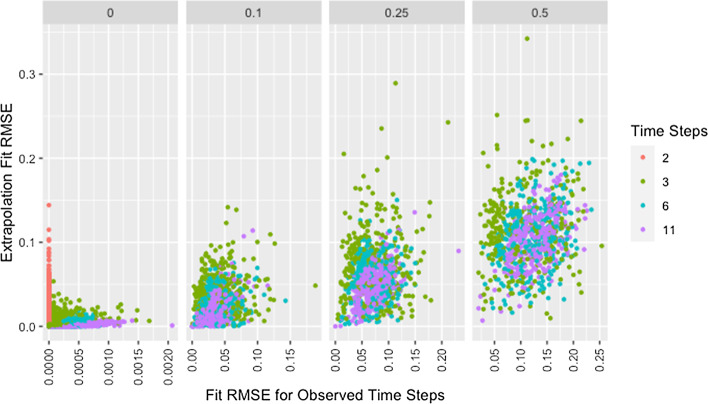
Extrapolation RMSE by fit RMSE, time steps, and missingness. RMSE for observed and predicted opinions on extrapolated time steps by RMSE for observed and predicted opinions on observed time steps, number of time steps, and proportion of missing data across panels with different x-axis scales

**Fig. 14:**
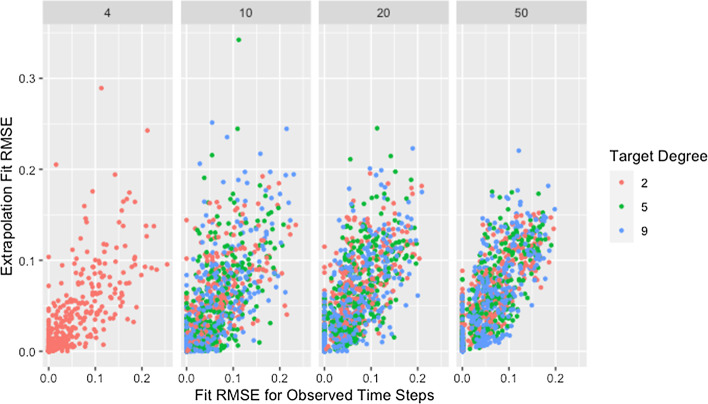
Extrapolation RMSE by fit RMSE, degree, and size. RMSE for observed and predicted opinions on extrapolated time steps by RMSE for observed and predicted opinions on observed time steps, target degree, and network size across panels

**Fig. 15:**
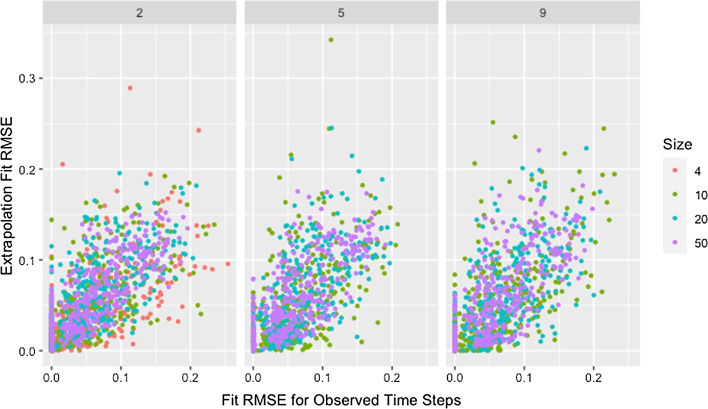
Extrapolation RMSE by fit RMSE, size, and degree. RMSE for observed and predicted opinions on extrapolated time steps by RMSE for observed and predicted opinions on observed time steps, network size, and target degree across panels

**Fig. 16:**
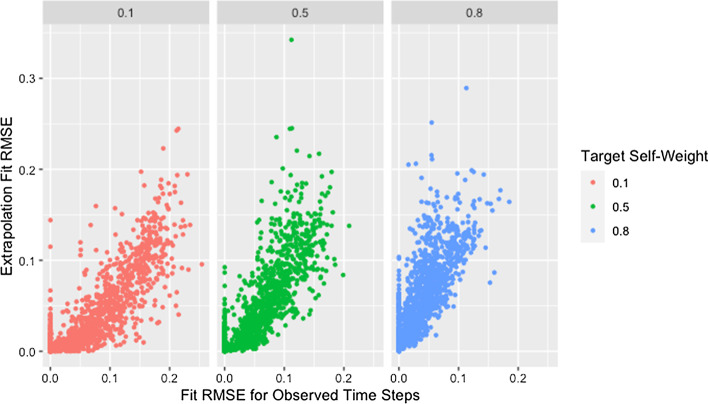
Extrapolation RMSE by fit RMSE and self-weight. RMSE for observed and predicted opinions on extrapolated time steps by RMSE for observed and predicted opinions on observed time steps and target self-weight

The collection of points in a vertical line for the simulations with only two time steps demonstrates the lack of a relationship which is consistent with worse parameter recovery for fewer time steps even when the estimated parameters fit the data well as was seen in Figs. 8 and 10. Fit on observed time steps is indicative of fit on extrapolated time steps across network size, degree, self-weight, and missingness as demonstrated by Figs. 13, 14, 15, and 16. The notable feature in all of these plots is the collection of points in a vertical line already discussed here.

## Application: diffusion of willingness to use $\text {PrEP}$ among $\text {BMSM}$

### Study overview

The pilot intervention study was conducted in Milwaukee, WI in 2016-2017 with social networks of $\text {BMSM}$ enrolled in the study. *Network leaders*—members of each network who were most socially interconnected with others in the same network, as well as those linked in their friendship with network members who would not otherwise be reached—were selected to attend a group intervention which met each week for five weeks for 2 h per session. Intervention sessions provided $\text {PrEP}$ education and skills training in how to endorse $\text {PrEP}$ to friends. All participants (network leaders and other network members) completed assessments at enrollment and three months later. The research was approved by the Medical College of Wisconsin Institutional Review Board (IRB), and written informed consent was provided by all study participants. Further information on procedures is available elsewhere [4].

#### Recruitment of social networks

Five social networks were enrolled in the study. Recruitment of each network began by identifying, approaching, and recruiting an initial *seed* in a community venue. Entry criteria for seeds were reporting male sex at birth; describing oneself as African American, Black, or multiracial; being age 18 or older; reporting sex with males in the past year; and not reporting being $\text {HIV}$ positive. When consented and enrolled in the study, seeds were asked to identify friends who were $\text {MSM}$. First names were recorded by the study staff, and the seed was provided with study invitation packets to distribute to listed friends. Interested friends of the seed were then enrolled following the same procedures as for the seed with the same entry criteria except not restricting study eligibility based on serostatus. This *first ring* of friends surrounding the seed were then asked to identify their own $\text {MSM}$ friends, and were asked to give an invitation packet to each named friend, with enrolled friends constituting the second and final ring extending outward from the seed. The recruited networks of the five seeds had a total of 40 unique members, and networks were composed of between 4 and 12 participating members.

#### Assessments

Assessment measures were completed using self-administered questionnaires during individual sessions at the time of the baseline and follow-up visits. Key measures for this analysis were $\text {PrEP}$ self-efficacy and $\text {PrEP}$ willingness. $\text {PrEP}$ self-efficacy was assessed with eight items. Each item asked participants to use a 4-point scale to indicate how difficult, from very hard to very easy, it would be to engage in an action (sample item: “How difficult or easy would it be for you to visit a doctor who can provide $\text {PrEP}$?”). $\text {PrEP}$ willingness was assessed with three items. Each item asked participants to indicate their strength of agreement using a 5-point Likert scale (from “strongly disagree” to “strongly agree”; sample item: “I would be willing to go on $\text {PrEP}$ if I had a casual sex partner who was $\text {HIV}$-positive”).

### Methods

We selected willingness and self-efficacy for this analysis since increasing willingness to take $\text {PrEP}$ is a key study aim and previous work has demonstrated a direct association between self-efficacy and $\text {PrEP}$ use [27]. A single value per agent was created for each measure by summing all the component Likert-scale items. Possible values ranged from 8-32 for self-efficacy and 3-15 for willingness. Since initial observations for both willingness and self-efficacy were missing for agent 11 in network 2 and agent 4 in network 3, we imputed these values using followup data for those agents where available or the median initial response for all other agents.

As the DeGroot model uses continuous data on the interval [0, 1], both scale measures were converted to continuous for model fitting and back-transformed for evaluation of model fit using the following processes:

#### Forward transformation

1. Begin with data on an *n*-point composite scale.
2. Divide the interval [0, 1] into *n* sub-intervals of equal width.
3. An opinion of *x* on the composite scale takes on the middle value, *y*, in the *x*th sub-interval on the continuous scale.

#### Back transformation

1. Begin with data on a continuous [0, 1] interval to be converted to an *n*-point composite scale.
2. Multiply the continuous opinion *y* by *n*.
3. Round the multiplied continuous opinion to an integer to produce an opinion on the composite scale.

The transformation to a continuous scale would allow us to use the objective function used for the simulation study,

$$\begin{aligned} f_C ({\hat{X}},X)=\sum _{i=1}^N\sum _{t=0}^{T-1}\big ({\hat{x}}_i (t)-x_i (t)\big )^2, \end{aligned}$$

but the optimal solution in the case of Likert-scale data is the one that best predicts observed opinions on the original scale. Specifically, predicted opinions that differ from the observed opinions on the continuous scale should only be penalized if they also differ on the original scale after the rounding step in the backwards transformation. We accomplish this through the use of the objective function

$$\begin{aligned} f_{L} ({\hat{X}},X)=\sum _{i=1}^N\sum _{t=0}^{T-1}B\big ({\hat{x}}_i (t),x_i (t)\big )\big |{\hat{x}}_i (t)-x_i (t)\big | \end{aligned}$$

where $B ({\hat{x}}_i (t),x_i (t))$ measures the absolute deviation between the observed and predicted opinions on the Likert scale. We refer to predicted opinions where $B ({\hat{x}}_i (t),x_i (t))=0$ as being in the correct *bin* or as a correctly predicted opinion. The inclusion of the absolute deviation on the continuous scale serves to penalize only estimates outside of the correct bin. Since the absolute deviation on the Likert scale as measured by $B ({\hat{x}}_i (t),x_i (t))$ is already a second penalty for predicted and observed opinions that differ greatly, we do not square the absolute deviation on the continuous scale as in the continuous version of the objective function. The fit can be assessed on a row or agent level by only summing across time steps to obtain an agent-level value of the objective function using

$$\begin{aligned} f_C ({\hat{x}}_i,x_i)=\sum _{t=0}^{T-1}\big ({\hat{x}}_i (t)-x_i (t)\big )^2. \end{aligned}$$

While there are only two time steps, the followup assessment was conducted three months after the initial assessment, meaning agents likely engaged in multiple interactions between the initial and followup assessments. To address this, we define a time step to be one month and treat time steps $t=1$ and $t=2$ as missing for all agents. This approach allows for information and opinions shared by the network seed to spread to their friends who can then share the information with their friends instead of assuming information from the seed has not yet reached the second ring of recruitment.

Since the recruitment process does not result in complete information about connections within the network, we construct two different adjacency matrices for each network: one where we begin with a matrix of zeros and add only connections we can be certain exist (denoted *Build* in reporting of results) and another where we begin with a matrix of ones and remove any connections we are certain do not exist (denoted *Remove* in reporting of results).

For all four combinations of outcome measure and adjacency matrix construction, we run the algorithm ten times. Though mathematically derived error estimates for either parameter estimates or predicted opinions are impractical, conducting multiple runs of the algorithm allows us to generate estimates of algorithmic variability for both. While we do not present point estimates for opinions as part of our results, we include standard deviations for all estimated weights. In order to assess algorithmic variability and predictive performance on a network level, we use modified forms of the RMSE measures used for the simulation study adapted to account for the unknown model parameter values and scale data:

$$\begin{aligned} RMSE_{fit}=\sqrt{\frac{\sum _{i=1}^N\big (B ({\hat{x}}_i (3),x_i (3)\big )^2}{CN (T-1)}} \end{aligned}$$

and

$$\begin{aligned} RMSE_{alg}=\sqrt{\frac{\sum _{i=1}^N\sum _{j=1}^N\sum _{r=1}^{10} ({\bar{w}}_{ij}-{\hat{w}}_{ij,r})^2}{10\sum _{i=1}^N\sum _{j=1}^Na_{ij}}}=\sqrt{\frac{\sum _{i=1}^P ({\bar{w}}_p-{\hat{w}}_p)^2}{10P}} \end{aligned}$$

where *C* represents the number of possible bins and is included to allow comparison of variability in estimated weights between the willingness and self-efficacy measures, $\bar{w}_{ij}$ is the mean estimated weight agent *i* places on the opinion of agent *j*, and $\bar{w}_p$ is the *p*th weight not fixed at zero.

### Results

To provide context for the following results, we include Table 10 which identifies both the seed and leader (s) in each social network. Note that some networks had a higher density of network leaders (range: one-eighth to one-third of network members).

**Table 10:** Network leaders and seeds

Network 1 ($N=12$)		
Agent 1	Leader	Seed
Agent 2	Leader	
Agent 3	Leader	
Agent 4	Leader	
Network 2 ($N=11$)		
Agent 1	Leader	Seed
Agent 2	Leader	
Agent 3	Leader	
Network 3 ($N=8$)		
Agent 1	Leader	Seed
Network 4 ($N=5$)		
Agent 1		Seed
Agent 4	Leader	
Network 5 ($N=4$)		
Agent 1	Leader	Seed
Agent 2	Leader	

Table of agents who are either network leaders or seeds by network

#### Accuracy of predicted opinions

**Fig. 17:**
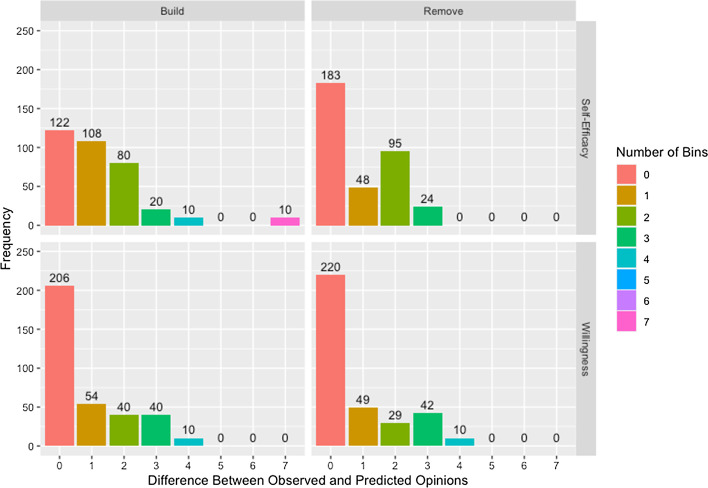
Difference between observed and predicted opinions by adjacency matrix and measure. Difference between observed and predicted opinions measured in number of bins by adjacency matrix construction method and measure across all networks and runs of the algorithm

We present Fig. 17 which demonstrates the models are able to predict opinions with reasonable accuracy while highlighting some limitations. It is worth noting that, while deviation is measured in number of *bins*—units on the composite scale—between observed and predicted opinions for both willingness and self-efficacy, the size of a bin is not the same between measures: a deviation of one bin is a larger difference for willingness than for self-efficacy.

The presence of the 10 observations that are seven bins away from the observed opinions using the build matrix for self-efficacy is particularly interesting. These observations are all from agent 5 in network 1. As can be seen in Table 11, agent 5 has only one connection in the build matrix, to agent 2. Since agent 2 has a lower self-efficacy score at both observed time steps than agent 5, their connection to agent 2 is unable to explain the improvement in self-efficacy at followup. In contrast, the model using the remove matrix has no deviations exceeding four bins. We see evidence of this effect across both measures with improved predicted opinions for the remove matrix. Table 11 also shows that agent 5 is allowed to update their opinion based on more agents within the network, explaining the improvement in predicted opinions. This is not to say that the remove matrix is a more accurate representation of the underlying network, only that information sources are missing from the build matrix. This could be a missing connection that is present in the remove matrix but it could also be a connection to an individual not included in the network or an external source such as social media, advertisements, or individual research.

**Table 11:** Estimated weights for willingness on network 1

Willingness build											
0.22* (0.23)	0.03* (0.03)	0.15* (0.10)	0.59* (0.12)	0	0	0	0	0	0	0	0
0.06* (0.04)	0.53* (0.10)	0	0	0.05 (0.04)	0.01 (0.00)	0.25 (0.10)	0.09 (0.06)	0.01 (0.01)	0	0	0
0.09* (0.14)	0	0.74* (0.03)	0	0	0	0	0	0	0.00 (0.00)	0.17 (0.12)	0
0.00* (0.00)	0	0	0.50* (0.24)	0	0	0	0	0	0	0	0.50 (0.24)
0	0.24* (0.00)	0	0	0.76 (0.00)	0	0	0	0	0	0	0
0	0.12* (0.00)	0	0	0	0.88 (0.00)	0	0	0	0	0	0
0	0.00* (0.01)	0	0	0	0	1.00 (0.01)	0	0	0	0	0
0	1.00* (0.00)	0	0	0	0	0	0.00 (0.00)	0	0	0	0
0	0.22* (0.00)	0	0	0	0	0	0	0.78 (0.00)	0	0	0
0	0	0.03* (0.01)	0	0	0	0	0	0	0.97 (0.01)	0	0
0	0	0.00* (0.00)	0	0	0	0	0	0	0	1.00 (0.00)	0
0	0	0	0.44* (0.21)	0	0	0	0	0	0	0	0.56 (0.21)
Willingness remove											
0.10* (0.07)	0.33* (0.08)	0.22* (0.02)	0.35* (0.07)	0	0	0	0	0	0	0	0
0.00* (0.00)	0.50* (0.42)	0.00* (0.00)	0.13* (0.25)	0.00 (0.00)	0.00 (0.00)	0.12 (0.24)	0.00 (0.00)	0.00 (0.00)	*0*.*00* (0.00)	*0*.*20* (0.35)	*0*.*05* (0.08)
0.03* (0.03)	0.02* (0.02)	0.71* (0.04)	0.02* (0.02)	*0*.*04* (0.05)	*0*.*00* (0.00)	*0*.*04* (0.05)	*0*.*02* (0.03)	*0*.*01* (0.02)	0.01 (0.02)	0.06 (0.07)	*0*.*04* (0.06)
0.00* (0.00)	0.18* (0.34)	0.00* (0.00)	0.28* (0.38)	*0*.*00* (0.00)	*0*.*00* (0.00)	*0*.*05* (0.06)	*0*.*00* (0.00)	*0*.*00* (0.00)	*0*.*00* (0.00)	*0*.*25* (0.35)	0.24 (0.36)
0	0.08* (0.11)	0.21* (0.09)	0.18* (0.17)	0.14 (0.18)	*0*.*00* (0.00)	*0*.*07* (0.06)	*0*.*03* (0.03)	*0*.*04* (0.04)	*0*.*00* (0.00)	*0*.*12* (0.09)	*0*.*13* (0.13)
0	0.05* (0.04)	0.22* (0.10)	0.04* (0.02)	*0*.*08* (0.10)	0.01 (0.01)	*0*.*10* (0.08)	*0*.*03* (0.03)	*0*.*36* (0.10)	*0*.*01* (0.01)	*0*.*05* (0.04)	*0*.*05* (0.03)
0	0.06* (0.11)	0.00* (0.00)	0.25* (0.28)	*0*.*00* (0.00)	*0*.*00* (0.00)	0.26 (0.29)	*0*.*20* (0.42)	*0*.*00* (0.00)	*0*.*00* (0.00)	*0*.*10* (0.10)	*0*.*14* (0.20)
0	0.01* (0.01)	0.00* (0.00)	0.01* (0.02)	*0*.*00* (0.00)	*0*.*02* (0.02)	*0*.*15* (0.31)	0.52 (0.43)	*0*.*00* (0.00)	*0*.*07* (0.05)	*0*.*02* (0.06)	*0*.*19* (0.33)
0	0.00* (0.00)	0.93* (0.10)	0.00* (0.00)	*0*.*00* (0.00)	*0*.*00* (0.00)	*0*.*00* (0.00)	*0*.*00* (0.00)	0.07 (0.11)	*0*.*00* (0.00)	*0*.*00* (0.00)	*0*.*00* (0.00)
0	0.01* (0.01)	0.02* (0.02)	0.01* (0.01)	*0*.*00* (0.01)	*0*.*07* (0.06)	*0*.*01* (0.01)	*0*.*02* (0.01)	*0*.*02* (0.02)	0.80 (0.10)	*0*.*02* (0.02)	*0*.*01* (0.02)
0	0.14* (0.27)	0.00* (0.00)	0.30* (0.34)	*0*.*00* (0.00)	*0*.*00* (0.00)	*0*.*12* (0.24)	*0*.*00* (0.00)	*0*.*00* (0.00)	*0*.*00* (0.00)	0.33 (0.41)	*0*.*11* (0.14)
0	0.09* (0.18)	0.00* (0.00)	0.04* (0.05)	*0*.*00* (0.00)	*0*.*00* (0.00)	*0*.*17* (0.25)	*0*.*20* (0.42)	*0*.*00* (0.00)	*0*.*00* (0.00)	*0*.*19* (0.36)	0.31 (0.42)

Estimated weights and standard deviations for willingness across 10 runs for network 1 using network built with connections known to exist and network where connections known not to exist are removed where weights placed on leaders is indicated with * and italic estimated weights in the remove matrix which are fixed in the build matrix

#### Weight estimates and variability

We present Tables 11, 12, 13, 14, 15, 16, 17 to summarize the estimated weights for all five networks across ten runs of the algorithm for both willingness and self-efficacy on adjacency matrices built from known ties or created by removing connections known not to exist. Elements fixed at 0 according to the adjacency matrix are indicated with a 0 without a standard deviation. Weights places on leaders are indicated with an asterisk (*) and weights that are structurally zero in the build matrix are italic in the remove matrix. As was discussed previously, there are notable differences in Table 11 for agent 5 between the build and remove matrices with estimated weights between 0.12 and 0.21 placed on agents whose weights were fixed at 0 for the build matrix. We also see evidence that the algorithm is able to identify connections present in the adjacency matrix that may not be present in the network in the form of zero or nearly zero estimated weights for both build and remove adjacency matrices.

**Table 12:** Estimated weights for self-efficacy on network 1

Efficacy build											
0.79* (0.02)	0.00* (0.00)	0.20* (0.03)	0.01* (0.04)	0	0	0	0	0	0	0	0
0.16* (0.04)	0.83* (0.02)	0	0	0.00 (0.00)	0.00 (0.00)	0.01 (0.02)	0.00 (0.00)	0.00 (0.00)	0	0	0
0.00* (0.00)	0	0.92* (0.01)	0	0	0	0	0	0	0.07 (0.02)	0.01 (0.01)	0
0.05* (0.05)	0	0	0.51* (0.19)	0	0	0	0	0	0	0	0.44 (0.14)
0	0.00* (0.00)	0	0	1.00 (0.00)	0	0	0	0	0	0	0
0	0.04* (0.00)	0	0	0	0.96 (0.00)	0	0	0	0	0	0
0	0.00* (0.00)	0	0	0	0	1.00 (0.00)	0	0	0	0	0
0	0.00* (0.00)	0	0	0	0	0	1.00 (0.00)	0	0	0	0
0	0.05* (0.00)	0	0	0	0	0	0	0.95 (0.00)	0	0	0
0	0	0.01* (0.01)	0	0	0	0	0	0	0.99 (0.01)	0	0
0	0	0.14* (0.00)	0	0	0	0	0	0	0	0.86 (0.00)	0
0	0	0	0.00* (0.00)	0	0	0	0	0	0	0	1.00 (0.00)
Efficacy remove											
0.70* (0.14)	0.02* (0.04)	0.09* (0.10)	0.19* (0.20)	0	0	0	0	0	0	0	0
0.02* (0.03)	0.69* (0.04)	0.00* (0.00)	0.01* (0.01)	0.27 (0.09)	0.00 (0.00)	0.00 (0.00)	0.00 (0.00)	0.00 (0.00)	*0*.*00* (0.00)	*0*.*01* (0.01)	*0*.*00* (0.00)
0.00* (0.00)	0.00* (0.00)	0.36* (0.09)	0.00* (0.00)	*0*.*00* (0.00)	*0*.*31* (0.08)	*0*.*31* (0.11)	*0*.*00* (0.00)	*0*.*00* (0.01)	0.01 (0.01)	0.00 (0.00)	*0*.*01* (0.01)
0.12* (0.13)	0.01* (0.01)	0.05* (0.06)	0.24* (0.28)	*0*.*03* (0.03)	*0*.*03* (0.03)	*0*.*23* (0.15)	*0*.*01* (0.01)	*0*.*00* (0.00)	*0*.*20* (0.33)	*0*.*03* (0.03)	0.06 (0.05)
0	0.03* (0.09)	0.03* (0.02)	0.11* (0.13)	0.69 (0.14)	*0*.*02* (0.01)	*0*.*03* (0.02)	*0*.*00* (0.00)	*0*.*01* (0.01)	*0*.*03* (0.03)	*0*.*01* (0.03)	*0*.*06* (0.06)
0	0.00* (0.01)	0.00* (0.01)	0.00* (0.00)	*0*.*00* (0.01)	0.77 (0.07)	*0*.*00* (0.00)	*0*.*01* (0.02)	*0*.*20* (0.11)	*0*.*01* (0.02)	*0*.*00* (0.00)	*0*.*00* (0.00)
0	0.00* (0.00)	0.00* (0.00)	0.00* (0.00)	*0*.*00* (0.00)	*0*.*01* (0.03)	0.99 (0.03)	*0*.*00* (0.00)	*0*.*00* (0.00)	*0*.*00* (0.00)	*0*.*00* (0.00)	*0*.*00* (0.00)
0	0.05* (0.03)	0.00* (0.01)	0.00* (0.00)	*0*.*13* (0.06)	*0*.*00* (0.01)	*0*.*00* (0.00)	0.8 (0.05)	*0*.*00* (0.01)	*0*.*00* (0.01)	*0*.*00* (0.01)	*0*.*00* (0.00)
0	0.01* (0.02)	0.00* (0.01)	0.00* (0.00)	*0*.*00* (0.00)	*0*.*01* (0.04)	*0*.*00* (0.00)	*0*.*11* (0.02)	0.86 (0.06)	*0*.*00* (0.00)	*0*.*00* (0.01)	*0*.*00* (0.00)
0	0.00* (0.01)	0.05* (0.07)	0.02* (0.04)	*0*.*00* (0.01)	*0*.*05* (0.06)	*0*.*04* (0.04)	*0*.*01* (0.01)	*0*.*03* (0.06)	0.04 (0.07)	*0*.*00* (0.00)	*0*.*74* (0.29)
0	0.06* (0.05)	0.05* (0.05)	0.15* (0.11)	*0*.*41* (0.13)	*0*.*02* (0.03)	*0*.*10* (0.12)	*0*.*00* (0.00)	*0*.*00* (0.00)	*0*.*12* (0.09)	0.05 (0.06)	*0*.*05* (0.05)
0	0.01* (0.01)	0.15* (0.10)	0.05* (0.06)	*0*.*01* (0.01)	*0*.*08* (0.05)	*0*.*19* (0.14)	*0*.*01* (0.01)	*0*.*02* (0.02)	*0*.*27* (0.16)	*0*.*03* (0.04)	0.17 (0.19)

Estimated weights and standard deviations for self-efficacy across 10 runs for network 1 using network built with connections known to exist and network where connections known not to exist are removed where weights placed on leaders is indicated with * and italic estimated weights in the remove matrix which are fixed in the build matrix

**Table 13:** Estimated weights for willingness on network 2

Willingness build										
0.99* (0.01)	0.00* (0.00)	0.00* (0.00)	0.00 (0.00)	0	0	0	0	0	0	0
0.18* (0.07)	0.82* (0.07)	0	0	0	0	0	0	0	0	0
0.23* (0.12)	0	0.02* (0.03)	0	0.17 (0.17)	0.11 (0.07)	0.16 (0.14)	0.07 (0.06)	0.24 (0.19)	0	0
0.50* (0.01)	0	0	0.02 (0.02)	0	0	0	0	0	0.00 (0.00)	0.48 (0.01)
0	0	0.18* (0.27)	0	0.82 (0.27)	0	0	0	0	0	0
0	0	0.00* (0.00)	0	0	1.00 (0.00)	0	0	0	0	0
0	0	0.24* (0.25)	0	0	0	0.76 (0.25)	0	0	0	0
0	0	0.23* (0.19)	0	0	0	0	0.77 (0.19)	0	0	0
0	0	0.10* (0.14)	0	0	0	0	0	0.90 (0.14)	0	0
0	0	0	1.00 (0.00)	0	0	0	0	0	0.00 (0.00)	0
0	0	0	0.00 (0.00)	0	0	0	0	0	0	1.00 (0.00)
Willingness Remove										
1.00* (0.00)	0.00* (0.00)	0.00* (0.00)	0.00 (0.00)	0	0	0	0	0	0	0
0.07* (0.08)	0.06* (0.08)	0.19* (0.31)	*0*.*03* (0.07)	*0*.*16* (0.26)	*0*.*03* (0.03)	*0*.*04* (0.08)	*0*.*19* (0.26)	*0*.*04* (0.05)	*0*.*03* (0.04)	*0*.*17* (0.29)
0.11* (0.15)	0.04* (0.06)	0.05* (0.08)	*0*.*31* (0.36)	0.06 (0.08)	0.05 (0.07)	0.10 (0.14)	0.14 (0.21)	0.09 (0.10)	*0*.*01* (0.02)	*0*.*03* (0.05)
0.06* (0.08)	0.13* (0.28)	0.20* (0.38)	0.06 (0.11)	*0*.*05* (0.07)	*0*.*04* (0.06)	*0*.*07* (0.15)	*0*.*15* (0.23)	*0*.*08* (0.18)	0.13 (0.31)	0.03 (0.04)
0	0.12* (0.20)	0.18* (0.27)	*0*.*17* (0.30)	0.09 (0.24)	*0*.*02* (0.03)	*0*.*03* (0.04)	*0*.*06* (0.09)	*0*.*06* (0.13)	*0*.*18* (0.30)	*0*.*08* (0.15)
0	0.00* (0.00)	0.00* (0.00)	*0*.*00* (0.00)	*0*.*00* (0.00)	1.00 (0.00)	*0*.*00* (0.00)	*0*.*00* (0.00)	*0*.*00* (0.00)	*0*.*00* (0.00)	*0*.*00* (0.00)
0	0.13* (0.16)	0.09* (0.10)	*0*.*19* (0.37)	*0*.*13* (0.19)	*0*.*04* (0.04)	0.04 (0.04)	*0*.*06* (0.06)	*0*.*09* (0.16)	*0*.*05* (0.05)	*0*.*19* (0.30)
0	0.07* (0.09)	0.22* (0.38)	*0*.*14* (0.28)	*0*.*05* (0.10)	*0*.*02* (0.03)	*0*.*11* (0.25)	0.05 (0.08)	*0*.*11* (0.16)	*0*.*17* (0.30)	*0*.*06* (0.07)
0	0.11* (0.10)	0.06* (0.07)	*0*.*14* (0.26)	*0*.*08* (0.10)	*0*.*04* (0.04)	*0*.*03* (0.04)	*0*.*17* (0.25)	0.13 (0.19)	*0*.*14* (0.31)	*0*.*09* (0.17)
0	0.01* (0.03)	0.00* (0.01)	0.02 (0.04)	*0*.*05* (0.09)	*0*.*45* (0.12)	*0*.*01* (0.01)	*0*.*08* (0.12)	*0*.*02* (0.05)	0.35 (0.25)	*0*.*00* (0.01)
0	0.14* (0.15)	0.05* (0.06)	0.17 (0.27)	*0*.*12* (0.27)	*0*.*03* (0.04)	*0*.*10* (0.18)	*0*.*08* (0.10)	*0*.*10* (0.11)	*0*.*05* (0.06)	0.14 (0.26)

Estimated weights and standard deviations for willingness across 10 runs for network 2 using network built with connections known to exist and network where connections known not to exist are removed where weights placed on leaders is indicated with * and italic estimated weights in the remove matrix which are fixed in the build matrix

**Table 14:** Estimated weights for self-efficacy on network 2

Self-efficacy build										
0.80* (0.01)	0.01* (0.01)	0.18* (0.02)	0.00 (0.00)	0	0	0	0	0	0	0
0.00* (0.00)	1.00* (0.00)	0	0	0	0	0	0	0	0	0
0.02* (0.02)	0	0.00* (0.00)	0	0.03 (0.05)	0.61 (0.08)	0.12 (0.13)	0.15 (0.14)	0.07 (0.08)	0	0
0.01* (0.01)	0	0	0.89 (0.02)	0	0	0	0	0	0.06 (0.03)	0.03 (0.02)
0	0	0.31* (0.01)	0	0.69 (0.01)	0	0	0	0	0	0
0	0	1.00* (0.00)	0	0	0.00 (0.00)	0	0	0	0	0
0	0	0.02* (0.04)	0	0	0	0.98 (0.04)	0	0	0	0
0	0	0.11* (0.01)	0	0	0	0	0.89 (0.01)	0	0	0
0	0	0.29* (0.01)	0	0	0	0	0	0.71 (0.01)	0	0
0	0	0	0.35 (0.01)	0	0	0	0	0	0.65 (0.01)	0
0	0	0	0.04 (0.02)	0	0	0	0	0	0	0.96 (0.02)
Self-efficacy remove										
1.00* (0.00)	0.00* (0.00)	0.00* (0.00)	0.00 (0.00)	0	0	0	0	0	0	0
0.07* (0.08)	0.06* (0.08)	0.19* (0.31)	*0*.*03* (0.07)	*0*.*16* (0.26)	*0*.*03* (0.03)	*0*.*04* (0.08)	*0*.*19* (0.26)	*0*.*04* (0.05)	*0*.*03* (0.04)	*0*.*17* (0.29)
0.84* (0.00)	0.16* (0.01)	0.00* (0.00)	0.00 (0.01)	0	0	0	0	0	0	0
0.02* (0.04)	0.01* (0.04)	0.00* (0.00)	*0*.*09* (0.11)	*0*.*01* (0.02)	*0*.*02* (0.02)	*0*.*03* (0.02)	*0*.*14* (0.13)	*0*.*02* (0.02)	*0*.*64* (0.31)	*0*.*01* (0.02)
0.09* (0.03)	0.06* (0.04)	0.20* (0.17)	*0*.*06* (0.06)	0.10 (0.03)	0.08 (0.10)	0.04 (0.04)	0.03 (0.02)	0.05 (0.02)	*0*.*03* (0.02)	*0*.*27* (0.14)
0.01* (0.01)	0.02* (0.04)	0.01* (0.02)	0.02 (0.04)	*0*.*01* (0.01)	*0*.*00* (0.01)	*0*.*03* (0.06)	*0*.*45* (0.32)	*0*.*40* (0.31)	0.02 (0.03)	0.02 (0.02)
0	0.00* (0.00)	0.00* (0.00)	*0*.*00* (0.00)	0.50 (0.01)	*0*.*50* (0.01)	*0*.*00* (0.00)	*0*.*00* (0.00)	*0*.*00* (0.00)	*0*.*00* (0.00)	*0*.*00* (0.00)
0	0.10* (0.13)	0.00* (0.00)	*0*.*08* (0.10)	*0*.*02* (0.04)	0.63 (0.09)	*0*.*09* (0.07)	*0*.*02* (0.02)	*0*.*01* (0.02)	*0*.*06* (0.05)	*0*.*00* (0.00)
0	0.00* (0.00)	0.00* (0.00)	*0*.*22* (0.41)	*0*.*00* (0.00)	*0*.*00* (0.00)	0.06 (0.19)	*0*.*67* (0.47)	*0*.*05* (0.12)	*0*.*00* (0.01)	*0*.*00* (0.00)
0	0.03* (0.06)	0.05* (0.05)	*0*.*10* (0.16)	*0*.*02* (0.02)	*0*.*02* (0.03)	*0*.*02* (0.04)	0.02 (0.06)	*0*.*68* (0.23)	*0*.*01* (0.02)	*0*.*04* (0.05)
0	0.00* (0.00)	0.07* (0.08)	*0*.*00* (0.00)	*0*.*02* (0.03)	*0*.*01* (0.02)	*0*.*00* (0.00)	*0*.*08* (0.26)	0.71 (0.25)	*0*.*00* (0.00)	*0*.*11* (0.06)
0	0.21* (0.12)	0.02* (0.02)	0.16 (0.12)	*0*.*02* (0.02)	*0*.*03* (0.03)	*0*.*21* (0.09)	*0*.*14* (0.14)	*0*.*06* (0.04)	0.11 (0.07)	*0*.*02* (0.02)

Estimated weights and standard deviations for self-efficacy across 10 runs for network 2 using network built with connections known to exist and network where connections known not to exist are removed where weights placed on leaders is indicated with * and italic estimated weights in the remove matrix which are fixed in the build matrix

**Table 15:** Estimated weights for willingness and self-efficacy on network 3

Willingness build							
0.05* (0.08)	0.00 (0.00)	0.49 (0.01)	0.46 (0.07)	0	0	0	0
1.00* (0.00)	0.00 (0.00)	0	0	0.00 (0.00)	0	0	0
0.04* (0.06)	0	0.96 (0.06)	0	0	0	0	0
0.00* (0.00)	0	0	0.15 (0.13)	0	0.51 (0.24)	0.34 (0.21)	0.00 (0.00)
0	0.51 (0.15)	0	0	0.49 (0.15)	0	0	0
0	0	0	0.53 (0.05)	0	0.47 (0.05)	0	0
0	0	0	0.49 (0.05)	0	0	0.51 (0.05)	0
0	0	0	0.24 (0.00)	0	0	0	0.76 (0.00)
Willingness remove							
0.02* (0.03)	0.94 (0.15)	0.01 (0.02)	0.03 (0.09)	0	0	0	0
0.25* (0.24)	0.44 (0.34)	*0*.*04* (0.05)	*0*.*11* (0.31)	0.03 (0.03)	*0*.*06* (0.10)	*0*.*03* (0.03)	*0*.*03* (0.01)
0.30* (0.10)	*0*.*05* (0.06)	0.10 (0.11)	*0*.*17* (0.15)	*0*.*13* (0.07)	*0*.*12* (0.08)	*0*.*07* (0.05)	*0*.*07* (0.01)
0.20* (0.27)	*0*.*01* (0.02)	*0*.*00* (0.00)	0.28 (0.29)	*0*.*16* (0.30)	0.13 (0.16)	0.22 (0.31)	0.00 (0.00)
0	0.26 (0.41)	*0*.*00* (0.00)	*0*.*04* (0.06)	0.12 (0.16)	*0*.*30* (0.32)	*0*.*29* (0.33)	*0*.*01* (0.02)
0	*0*.*00* (0.00)	*0*.*00* (0.00)	0.44 (0.39)	*0*.*08* (0.20)	0.20 (0.29)	*0*.*28* (0.36)	*0*.*00* (0.00)
0	*0*.*03* (0.07)	*0*.*00* (0.00)	0.15 (0.24)	*0*.*10* (0.17)	*0*.*40* (0.33)	0.32 (0.32)	*0*.*00* (0.00)
0	*0*.*00* (0.00)	*0*.*20* (0.07)	0.02 (0.02)	*0*.*01* (0.04)	*0*.*01* (0.01)	*0*.*02* (0.06)	0.74 (0.01)
Self-efficacy build							
0.96* (0.04)	0.03 (0.03)	0.00 (0.00)	0.01 (0.02)	0	0	0	0
0.00* (0.00)	0.94 (0.01)	0	0	0.06 (0.01)	0	0	0
0.14* (0.00)	0	0.86 (0.00)	0	0	0	0	0
0.00* (0.00)	0	0	0.93 (0.10)	0	0.06 (0.10)	0.00 (0.01)	0.01 (0.01)
0	0.01 (0.02)	0	0	0.99 (0.02)	0	0	0
0	0	0	0.31 (0.03)	0	0.69 (0.03)	0	0
0	0	0	0.00 (0.00)	0	0	1.00 (0.00)	0
0	0	0	0.14 (0.00)	0	0	0	0.86 (0.00)
Self-efficacy remove							
0.91* (0.02)	0.05 (0.02)	0.00 (0.00)	0.03 (0.03)	0	0	0	0
0.02* (0.01)	0.67 (0.24)	*0*.*06* (0.01)	*0*.*14* (0.25)	0.03 (0.03)	*0*.*05* (0.06)	*0*.*02* (0.01)	*0*.*02* (0.01)
0.00* (0.01)	*0*.*00* (0.00)	0.85 (0.01)	*0*.*03* (0.06)	*0*.*01* (0.01)	*0*.*00* (0.00)	*0*.*10* (0.06)	*0*.*00* (0.01)
0.03* (0.05)	*0*.*26* (0.35)	*0*.*02* (0.03)	0.04 (0.09)	*0*.*48* (0.38)	0.06 (0.07)	0.08 (0.12)	0.02 (0.01)
0	0.04 (0.02)	*0*.*03* (0.03)	*0*.*13* (0.12)	0.34 (0.15)	*0*.*13* (0.13)	*0*.*18* (0.10)	*0*.*15* (0.15)
0	*0*.*18* (0.06)	*0*.*01* (0.01)	0.08 (0.05)	*0*.*04* (0.03)	0.52 (0.15)	*0*.*06* (0.04)	*0*.*12* (0.04)
0	*0*.*00* (0.00)	*0*.*00* (0.00)	0.00 (0.00)	*0*.*00* (0.01)	*0*.*00* (0.00)	0.64 (0.02)	*0*.*35* (0.01)
0	*0*.*02* (0.01)	*0*.*00* (0.00)	0.01 (0.01)	*0*.*01* (0.01)	*0*.*07* (0.04)	*0*.*02* (0.03)	0.87 (0.03)

Estimated weights and standard deviations for willingness and self-efficacy across 10 runs for network 3 using network built with connections known to exist and network where connections known not to exist are removed where weights placed on leaders is indicated with * and italic estimated weights in the remove matrix which are fixed in the build matrix

**Table 16:** Estimated weights for willingness and self-efficacy on network 4

Build					Remove				
Willingness									
0.63 (0.05)	0.01 (0.01)	0.36 (0.05)	0.00* (0.00)	0	0.4 (0.21)	0.01 (0.01)	0.57 (0.20)	0.02* (0.02)	0
0.28 (0.00)	0.72 (0.00)	0	0	0	0.18 (0.08)	0.70 (0.04)	*0*.*06* (0.06)	0.01* (0.02)	*0*.*04* (0.06)
0.00 (0.00)	0	1.00 (0.00)	0	0	0.00 (0.01)	*0*.*00* (0.00)	0.99 (0.02)	0.00* (0.00)	*0*.*00* (0.01)
0.17 (0.00)	0	0	0.83* (0.00)	0.00 (0.00)	0.00 (0.00)	*0*.*32* (0.00)	*0*.*00* (0.00)	0.68* (0.00)	0.00 (0.00)
0	0	0	0.00* (0.01)	1.00 (0.01)	0	*0*.*01* (0.01)	*0*.*61* (0.28)	0.03* (0.02)	0.35 (0.31)

Self-efficacy									
0.00 (0.00)	0.87 (0.00)	0.00 (0.00)	0.13* (0.00)	0	0.72 (0.06)	0.08 (0.02)	0.15 (0.04)	0.04* (0.03)	0
1.00 (0.00)	0.00 (0.00)	0	0	0	0.00 (0.00)	0.70 (0.01)	*0*.*00* (0.00)	0.25* (0.04)	*0*.*05* (0.05)
0.03 (0.02)	0	0.97 (0.02)	0	0	0.01 (0.01)	*0*.*00* (0.00)	0.99 (0.01)	0.00* (0.00)	*0*.*00* (0.00)
0.00 (0.00)	0	0	1.00* (0.00)	0.00 (0.00)	0.00 (0.00)	*0*.*00* (0.00)	*0*.*00* (0.00)	1.00* (0.00)	0.00 (0.00)
0	0	0	0.10* (0.04)	0.90 (0.04)	0	*0*.*00* (0.00)	*0*.*07* (0.00)	0.91* (0.03)	0.02 (0.03)

Estimated weights and standard deviations for willingness and self-efficacy across 10 runs for network 4 using network built with connections known to exist and network where connections known not to exist are removed where weights placed on leaders is indicated with * and italic estimated weights in the remove matrix which are fixed in the build matrix

**Table 17:** Estimated weights for willingness and self-efficacy on network 5

Build				Remove			
Willingness							
0.50* (0.09)	0.50* (0.09)	0.00 (0.00)	0.00 (0.00)	0.48* (0.06)	0.52* (0.06)	0.00 (0.00)	0.00 (0.00)
0.49* (0.06)	0.51* (0.06)	0	0	0.53* (0.14)	0.47* (0.14)	*0*.*00* (0.00)	*0*.*00* (0.00)
0.04* (0.00)	0	0.96 (0.00)	0	0.00* (0.00)	0.00* (0.00)	0.68 (0.01)	*0*.*32* (0.01)
0.21* (0.00)	0	0	0.79 (0.00)	0.12* (0.07)	0.09* (0.07)	*0*.*00* (0.00)	0.79 (0.00)

Self-efficacy							
0.71* (0.00)	0.00* (0.00)	0.00 (0.00)	0.29 (0.00)	0.71* (0.00)	0.00* (0.00)	0.00 (0.00)	0.29 (0.00)
0.12* (0.00)	0.88* (0.00)	0	0	0.10* (0.03)	0.88* (0.01)	*0*.*00* (0.01)	*0*.*02* (0.03)
0.00* (0.00)	0	1.00 (0.00)	0	0.00* (0.00)	0.00* (0.00)	1.00 (0.00)	*0*.*00* (0.00)
0.00* (0.00)	0	0	1.00 (0.00)	0.00* (0.00)	0.00* (0.00)	*0*.*00* (0.00)	1.00 (0.00)

Estimated weights and standard deviations for willingness and self-efficacy across 10 runs for network 5 using network built with connections known to exist and network where connections known not to exist are removed

While Tables 11, 12, 13, 14, 15, 16, 17 do provide information about variability between runs of the algorithm, Table 18 presents this information in condensed form. It shows that algorithmic variability is much lower for self-efficacy than for willingness. Within measures, algorithmic variability is higher on the remove matrix for willingness and higher on the build matrix for self-efficacy. The assessment of how well the predicted opinions fit the data confirms the relationship seen in Fig. 17 with models on the remove matrix tending to produce predicted opinions closer to those observed. Again, this supports the idea that information is missing from the build matrices though the additional information in the remove matrices is not necessarily correct.

**Table 18:** Mean self-weight, mean weight, proportion, and number by network, adjacency matrix and leadership for self-efficacy

Network	Measure	Adjacency	${\mathbf{RMSE}}_{{{\mathbf{alg}}}}$	${\mathbf{RMSE}}_{{{\mathbf{fit}}}}$
1 ($N=12$)	Willingness	Build	0.09	0.36 (0.01)
		Remove	0.16	0.37 (0.02)
	Self-efficacy	Build	0.04	0.51 (0.00)
		Remove	0.00	0.28 (0.02)
2 ($N=11$)	Willingness	Build	0.12	0.45 (0.00)
		Remove	0.19	0.42 (0.01)
	Self-efficacy	Build	0.04	0.23 (0.00)
		Remove	0.00	0.23 (0.01)
3 ($N=8$)	Willingness	Build	0.09	0.33 (0.01)
		Remove	0.20	0.33 (0.00)
	Self-efficacy	Build	0.03	0.21 (0.01)
		Remove	0.00	0.15 (0.00)
4 ($N=5$)	Willingness	Build	0.02	0.71 (0.00)
		Remove	0.11	0.70 (0.03)
	Self-efficacy	Build	0.02	0.54 (0.00)
		Remove	0.00	0.42 (0.00)
5 ($N=4$)	Willingness	Build	0.05	0.32 (0.00)
		Remove	0.06	0.29 (0.00)
	Self-efficacy	Build	0.00	0.38 (0.00)
		Remove	0.00	0.38 (0.00)

Mean self-weight, mean weight from other agents, and proportion and number of possible weights from other agents that exceed 0.005 for self-efficacy

#### Comparison of leaders to other agents

In order to assess whether leaders are better able to influence their friends, we calculate the mean weight placed on leaders versus non-leaders for each network, adjacency matrix, and measure combination. We also determine, out of all possible non-zero weights placed on leaders or non-leaders, both the number and proportion that exceed 0.005 for all combinations. All of these measures exclude agents not connected to a leader. We also include mean self-weight for leaders and non-leaders for all agents within the networks. Table 19 shows these summary statistics for willingness.

**Table 19:** Mean self-weight, mean weight, proportion, and number by network, adjacency matrix and leadership for willingness

Network	Adjacency	Leader	Self-weight	Weight	Proportion	Number
1 ($N=12$)	Build	Leader	0.50 (0.21)	0.21 (0.29)	0.79	11
		Non-leader	0.74 (0.34)	0.14 (0.17)	0.88	7
	Remove	Leader	0.40 (0.26)	0.11 (0.18)	0.72	26
		Non-leader	0.30 (0.26)	0.05 (0.08)	0.55	44
2 ($N=11$)	Build	Leader	0.61 (0.52)	0.17 (0.15)	0.70	7
		Non-leader	0.66 (0.41)	0.15 (0.16)	0.75	6
	Remove	Leader	0.37 (0.55)	0.09 (0.07)	0.78	18
		Non-leader	0.23 (0.33)	0.08 (0.08)	0.88	64
3 ($N=8$)	Build	Leader	0.05 (NA)	0.35 (0.57)	0.67	2
		Non-leader	0.48 (0.33)	0.26 (0.25)	0.57	4
	Remove	Leader	0.02 (NA)	0.25 (0.05)	1.00	3
		Non-leader	0.31 (0.22)	0.11 (0.17)	0.80	36
4 ($N=5$)	Build	Leader	0.83 (NA)	0.00 (0.00)	0.00	0
		Non-leader	0.84 (0.19)	0.14 (0.16)	0.67	4
	Remove	Leader	0.68 (NA)	0.02 (0.01)	0.75	3
		Non-leader	0.61 (0.30)	0.13 (0.21)	0.57	8
5 ($N=4$)	Build	Leader	0.50 (0.01)	0.31 (0.23)	1.00	4
		Non-leader	0.88 (0.12)	0.00 (0.00)	0.00	0
	Remove	Leader	0.48 (0.01)	0.21 (0.25)	0.67	4
		Non-leader	0.74 (0.08)	0.05 (0.13)	0.17	1

Mean self-weight, mean weight from other agents, and proportion and number of possible weights from other agents that exceed 0.005 for willingness with standard deviations where applicable

For willingness, on networks 1, 2, 3, and 5 the mean estimated weight for leaders is higher for both adjacency matrices. It is higher for non-leaders across both adjacency matrices for network 4. There is no clear pattern in the proportion of practically non-zero weights (weights greater than 0.005) between leaders and non-leaders. We note that the uncertainty in the adjacency matrices could obscure any relationship between leadership training and the proportion of possible non-zero weights that are present and that network 4 is the only network where the seed did not attend leadership training, providing a possible explanation for the inefficacy of the leader in that network. For both measures, mean self-weight tends to be higher for the build matrix than for the remove matrix, indicating the algorithm identifies a solution where weight is distributed over either more or different agents when available.

Table 20 presents the same statistics for self-efficacy. For self-efficacy, whether higher weight was placed on leaders or non-leaders is only consistent between the build and bemove matrices for networks 1 and 5 with higher weight placed on non-leaders. With network 4 again being a notable exception, both the weights and the differences between them tend to be small relative to those estimated for willingness. This necessarily results in higher estimated self-weights than for willingness, indicating agents were more open to changing their willingness to use $\text {PrEP}$ than their beliefs about the difficulty of engaging in $\text {PrEP}$ behaviors. Again, there is no clear pattern in the proportion of practically non-zero weights between leaders and non-leaders and self-weight tends to be higher for the build matrix.

**Table 20:** Mean self-weight, mean weight, proportion, and number by network, adjacency matrix and leadership for self-efficacy

Network	Adjacency	Leader	Self-Weight	Weight	Proportion	Number
1 ($N=12$)	Build	Leader	0.76 (0.18)	0.05 (0.07)	0.57	8
		Non-leader	0.97 (0.05)	0.07 (0.15)	0.50	4
	Remove	Leader	0.50 (0.23)	0.04 (0.05)	0.58	21
		Non-leader	0.55 (0.39)	0.05 (0.12)	0.54	43
2 ($N=11$)	Build	Leader	0.60 (0.53)	0.20 (0.31)	0.90	9
		Non-leader	0.72 (0.32)	0.13 (0.20)	0.88	7
	Remove	Leader	0.35 (0.43)	0.04 (0.06)	0.57	13
		Non-leader	0.32 (0.29)	0.09 (0.16)	0.70	51
3 ($N=8$)	Build	Leader	0.96 (NA)	0.05 (0.08)	0.33	1
		Non-leader	0.90 (0.11)	0.02 (0.03)	0.71	5
	Remove	Leader	0.91 (NA)	0.02 (0.01)	0.67	2
		Non-leader	0.56 (0.29)	0.07 (0.10)	0.78	35
4 ($N=5$)	Build	Leader	1.00 (NA)	0.12 (0.02)	1.0	2
		Non-leader	0.47 (0.54)	0.22 (0.44)	0.25	1
	Remove	Leader	1.00 (NA)	0.30 (0.42)	0.75	3
		Non-leader	0.61 (0.41)	0.03 (0.05)	0.36	5
5 ($N=4$)	Build	Leader	0.80 (0.12)	0.03 (0.06)	0.25	1
		Non-leader	1.00 (0.00)	0.14 (0.21)	0.5	1
	Remove	Leader	0.80 (0.12)	0.02 (0.04)	0.17	1
		Non-leader	1.00 (0.00)	0.05 (0.12)	0.33	2

Mean self-weight, mean weight from other agents, and proportion and number of possible weights from other agents that exceed 0.005 for self-efficacy with standard deviations where applicable

## Conclusions

In order to expand the use of opinion diffusion models to public health and social science applications, we developed a novel genetic algorithm capable of recovering the parameters of a DeGroot opinion diffusion process using small data sets, including those with missing data and more model parameters than observed time steps. We assessed the efficacy of the algorithm on simulated data, considering a variety of features of the networks and data sets where this method could reasonably be used. We also demonstrated the performance of the algorithm on the $\text {PrEP}$ pilot study data, producing estimated weights that result in predicted opinions close to those observed. This serves as a first step in the development of an epidemic model informed by the opinion diffusion process to assess the network leader intervention, an option not previously available with the limited size of the data set.

### Simulation study

The simulation study demonstrates the algorithm is able to recover the model parameters of the opinion diffusion process and correctly predict opinions, though the accuracy of both types of estimates depend on degree, network size, self-weight, number of time steps observed, and proportion of missing data. Small, low degree networks are the only networks capable of nearly perfect model parameter recovery but can also result in inaccurate model parameter estimates even when the solution fits the observed opinions well. Increased network size mitigates this issue, decreasing both the median and IQR of the recovery RMSE at the expense of increasing the minimum recovery RMSE. Networks comprised of agents who place little weight on their own opinions also result in poorer recovery of weights even when predicted opinions closely match the observed ones. Low degree may exacerbate this problem but larger network size mitigates the effect of low self-weight as for low degree. Both lower proportions of missing data and more time steps improve recovery by increasing the number of observations per agent though applying the algorithm to data with a few completely observed time points will result in better parameter recovery than an application with a comparable number of observed time points per agent resulting from more follow-up time points but significant missing data.

### Opinion diffusion modeling

Analysis of the estimated weights demonstrates that agents generally place more weight on the opinions of leaders than non-leaders when updating their willingness to use $\text {PrEP}$, though evidence is mixed for self-efficacy. They also suggest the network leader intervention is more effective for changing willingness than self-efficacy. While these results agree with previous research and indicate the use of opinion leaders may be an effective intervention for increasing $\text {PrEP}$ usage to reduce transmission of $\text {HIV}$, this is not the intended use of the estimated weights. Instead, the application of this algorithm to the pilot study data is the first step in a detailed investigation of the opinion diffusion process underlying the use of network leaders as an intervention to increase uptake of $\text {PrEP}$ for $\text {BMSM}$.

Though other methods can be used to assess the effectiveness of the intervention for each agent, this method allows for an exploration of why the intervention was or was not effective for each agent in the network. Since the model includes an estimate of the weight each agent places on the opinions of all other agents, we are able to identify agents who were particularly receptive or resistant to the opinions of the network leaders. Identifying demographic or relational differences that explain the varying receptivity of agents with the network allows for better prediction of the change in opinions about $\text {PrEP}$ that could be expected when applying the network leader intervention to other networks.

While only having two time steps is a limitation that results in higher variability in model parameter estimates, both the simulation study and application of the algorithm to the pilot study data show the algorithm can be used to estimate the model parameters of the diffusion process using very few time points. In addition, the simulation study demonstrates a substantial improvement in the performance of the algorithm on three time steps as compared to two time steps. This suggests the algorithm will be able to produce estimates that are more precise and accurate using the three time steps included in the full study. These estimates can then be used to inform parameters of an epidemic model for evaluating the use of a network leader intervention as a means to reduce incidence of $\text {HIV}$ for $\text {BMSM}$.

## Data Availability

The data generated and analysed during the simulation study are available in the corresponding author’s GitHub repository, https://github.com/karajohnson4/DeGrootGeneticAlgorithm. The data from the pilot study are available from Jeffrey A. Kelly, PhD, cairdirector%40mcw.edu but restrictions apply to the availability of these data, which were used under license for the current study, and so are not publicly available. Data are however available from the authors upon reasonable request and with permission of Jeffrey A. Kelly, cairdirector%40mcw.edu. The genetic algorithm code is also available in the corresponding author’s GitHub repository, https://github.com/karajohnson4/DeGrootGeneticAlgorithm under the name Algorithm-Code. The ANS-Archive branch will serve as an archived version. The code is written in Julia, is platform independent, requires Julia 1.5 or higher, and uses the GNU GENERAL PUBLIC LICENSE [26]. Analysis was performed in R using RStudio with data imported using haven and restructured using tidyr [28–31]. Plots were generated with ggplot2 [32].

## Abbreviations

BMSM: Black men who have sex with men
HIV: human immunodeficiency virus
IRB: Institutional Review Board
MSM: men who have sex with men
PrEP: pre-exposure prophylaxis
RMSE: root-mean-square error
SIR: Susceptible-infected-recovered
SODM: Stochastic opinion dynamics model

## Footnotes

1 Naive learning can be expressed as a DeGroot model with $w_{ij}=\frac{1}{N_i+1}$ for $a_{ij}\ne 0$ where $w_{ij}$ and $a_{ij}$ are as defined in the DeGroot Model subsection below and $N_i$ is the number of agents in the neighborhood of agent *i*.

2 Within this paper, the observed and predicted initial opinions are equal, meaning that the contribution to the value of the objective function and other fit assessments from opinions at time $t=0$ is always 0. For use of extensions to this method when observed and predicted initial opinions differ, we present all relevant equations in the form that incorporates the deviation between observed and predicted initial opinions.
